# Netrin-1 Protects Hepatocytes Against Cell Death Through Sustained Translation During the Unfolded Protein Response

**DOI:** 10.1016/j.jcmgh.2015.12.011

**Published:** 2016-01-09

**Authors:** Thomas Lahlali, Marie-Laure Plissonnier, Cristina Romero-López, Maud Michelet, Benjamin Ducarouge, Alfredo Berzal-Herranz, Fabien Zoulim, Patrick Mehlen, Romain Parent

**Affiliations:** 1 Inserm U1052-UMR CNRS 5286, Centre Léon Berard, Centre de Recherche en Cancérologie, Lyon, France; 3 Inserm U1052-UMR Centre National de la Recherche Scientifique 5286, Centre Léon Berard, Centre de Recherche en Cancérologie, Lyon, France; 2 Instituto de Parasitología y Biomedicina López-Neyra Consejo Superior de Investigaciones Científicas, Ciencia e Investigación (IPBLN-CSIC), Parque Tecnológico Ciencias de la Salud Granada, Armilla, Granada, Spain

**Keywords:** Netrin, UPR, Hepatocyte, Translation, ATF6, activated transcription factor 6, CMV, cytomegalovirus, DAPK, death-associated protein kinase, DMS, dimethyl sulfate, DR, dependence receptor, DTT, dithiothreitol, eIF2α, Eukaryotic translation initiation factor 2A, ER, endoplasmic reticulum, FLuc, Firefly luciferase, HBV, hepatitis B virus, HCC, hepatocellular carcinoma, HCV, hepatitis C virus, IRE1α, inositol requiring enzyme 1α, IRES, internal ribosome entry site, LSL, (Lox-Stop-Lox), mRNA, messenger RNA, NMIA, N-methyl-isatoic anhydride, pBic, Bicistronic plasmid, PBS, phosphate-buffered saline, PERK, protein kinase RNA (PKR)-like endoplasmic reticulum kinase, PP2A, protein phosphatase 2A, PR65β, erine/threonine-protein phosphatase 2A 65 kDa regulatory subunit A beta isoform, qRT-PCR, quantitative reverse-transcription polymerase chain reaction, RLuc, Renilla lucerifase, siRNA, small interfering RNA, Tu, tunicamycin, TUNEL, terminal deoxynucleotidyl transferase–mediated deoxyuridine triphosphate nick-end labeling, UNC5, uncoordinated phenotype-5, UPR, unfolded protein response, UTR, untranslated region, VR1, vanilloid receptor 1

## Abstract

**Background & Aims:**

Netrin-1, a multifunctional secreted protein, is up-regulated in cancer and inflammation. Netrin-1 blocks apoptosis induced by the prototypical dependence receptors deleted in colorectal carcinoma and uncoordinated phenotype-5. Although the unfolded protein response (UPR) triggers apoptosis on exposure to stress, it first attempts to restore endoplasmic reticulum homeostasis to foster cell survival. Importantly, UPR is implicated in chronic liver conditions including hepatic oncogenesis. Netrin-1's implication in cell survival on UPR in this context is unknown.

**Methods:**

Isolation of translational complexes, determination of RNA secondary structures by selective 2’-hydroxyl acylation and primer extension/dimethyl sulfate, bicistronic constructs, as well as conventional cell biology and biochemistry approaches were used on in vitro–grown hepatocytic cells, wild-type, and netrin-1 transgenic mice.

**Results:**

HepaRG cells constitute a bona fide model for UPR studies in vitro through adequate activation of the 3 sensors of the UPR (protein kinase RNA–like endoplasmic reticulum kinase (PERK)), inositol requiring enzyme 1α (IRE1α), and activated transcription factor 6 (ATF6). The netrin-1 messenger RNA 5'-end was shown to fold into a complex double pseudoknot and bear E-loop motifs, both of which are representative hallmarks of related internal ribosome entry site regions. Cap-independent translation of netrin 5' untranslated region–driven luciferase was observed on UPR in vitro. Unlike several structurally related oncogenic transcripts (l-myc, c-myc, c-myb), netrin-1 messenger RNA was selected for translation during UPR both in human hepatocytes and in mice livers. Depletion of netrin-1 during UPR induces apoptosis, leading to cell death through an uncoordinated phenotype-5A/C–mediated involvement of protein phosphatase 2A and death-associated protein kinase 1 in vitro and in netrin transgenic mice.

**Conclusions:**

UPR-resistant, internal ribosome entry site–driven netrin-1 translation leads to the inhibition of uncoordinated phenotype-5/death-associated protein kinase 1–mediated apoptosis in the hepatic context during UPR, a hallmark of chronic liver disease.

**Summary**

The unfolded protein response (UPR) is a hallmark of numerous liver diseases including cancer. Here, we report that in the liver, netrin-1 protects against UPR-related cell death through UPR-resistant, internal ribosome entry site–driven translation, and the UNC5/death-associated protein kinase pathway.

The endoplasmic reticulum (ER) is the place of secretory and membrane protein synthesis.^1^ Folding of newly synthesized proteins within the ER lumen is tightly monitored by dedicated quality control machinery.^2^ Perturbation of ER homeostasis caused by hypoxia, viral infection, or other stressors can reduce or surpass the ER folding capacity, resulting in a condition termed *ER stress*.^3^ Chronic ER stress is observed in several diseases including cancer.^4^, ^5^, ^6^ Likewise, ER stress plays a well-documented role in hepatitis B virus (HBV)- and hepatitis C virus (HCV)-related pathogenesis,^7^, ^8^, ^9^ implicating it as a factor in liver disease and carcinogenesis.^7^, ^10^, ^11^, ^12^ To restore homeostasis in response to ER stress, cells activate the unfolded protein response (UPR), a process involving the sequential activation of 3 ER sensors named protein kinase RNA (PKR)-like endoplasmic reticulum kinase (PERK), activated transcription factor 6 (ATF6), and inositol requiring enzyme 1α (IRE1α).^13^ PERK phosphorylates the elongation factor Eukaryotic translation initiation factor 2A (eIF2α) at Ser-51, impeding protein translation. If the UPR fails to restore ER homeostasis, it instead reverts to apoptosis.^13^ One of the mediators of UPR-induced apoptosis is the death-associated protein kinase 1 (DAPK1), a key regulator of cell death.^14^ DAPK1 is activated by protein phosphatase 2A (PP2A) in a process that requires interactors including calmodulin, and, interestingly, the Unc-5 homolog B (UNC5B),^15^, ^16^ suggesting a potential involvement of extracellular or autocrine factors.

UNC5B is one of 4 members of the UNC5-family (UNC5A, UNC5B, UNC5C, and UNC5D), belonging to the so-called dependence receptors (DRs),^17^ promoting cell survival as long as they are engaged by their ligands. Once unbound, these receptors trigger apoptosis.^17^, ^18^, ^19^ Netrin-1 is the canonical soluble partner of DRs. It initially was identified as an axonal guidance molecule of the developing central nervous system.^20^ In the past decade, several studies have reported that netrin-1 is up-regulated in several types of cancer^19^, ^21^, ^22^, ^23^, ^24^ and cancer-associated inflammatory diseases conferring cells with a selective advantage regarding survival and proliferation.^25^, ^26^, ^27^ Netrin-1 is upregulated in cancers in general and in cancer-associated associated inflammatory diseases. Intriguingly, netrin-1 is increased by 10- to 30-fold upon HBV or HCV infection in an epidermal growth factor receptor–dependent manner in the latter case, and also in cirrhosis irrespective of its etiology (Plissonnier et al, unpublished data). From what is known, UNC5A and C induce apoptosis through the recruitment of neurotrophin receptor-interacting MAGE homolog or the activation of the E2F Transcription Factor 1 transcription factor, respectively.^15^, ^16^ As mentioned earlier, UNC5B binds and signals via DAPK1, triggering a signal cascade that has been well described. Briefly, in the presence of netrin-1, the UNC5B receptor interacts with an inactive, phosphorylated form of DAPK1. In the absence of netrin-1, UNC5B adopts an open conformation and recruits Serine/threonine-protein phosphatase 2A 65 kDa regulatory subunit A beta isoform (PR65β)/PP2A into an UNC5B/DAPK1 complex followed by caspase-3 activation.^16^ Two recent studies have suggested a link between netrin-1 and the UPR.^28^, ^29^ Given the already known association of UNC5B and DAPK1 with this process, we sought to investigate the role of netrin-1/UNC5-controlled apoptosis in UPR-associated cell death.

In the liver, common triggers include viral infections, alcoholic liver disease, or genetic conditions, all of which are high risk factors for hepatocellular carcinoma.^7^, ^8^, ^9^, ^30^, ^31^, ^32^ The UPR is a hallmark of these pathophysiological contexts. Here, we show that during experimentally induced UPR, netrin-1 is efficiently translated through an internal ribosome entry site (IRES) both in vitro and in vivo in mice livers. Modulation of netrin-1 in hepatocytic cells conditions caspase-3 activation and affects cell death via UNC5A- and UNC5C-mediated increase of PP2A activity and implication of DAPK1. Our results indicate that netrin-1 protects hepatocytes against UPR-related cell death through resistance to UPR-related global translational inhibition.

## Materials and Methods

### Cell Culture

HepaRG cells were cultured as previously described.^33^ The human hepatoma cell line Huh7.5 was grown in Dulbecco's modified Eagle medium (Life Technologies, Carlsbad, CA), supplemented with 10% fetal bovine serum (Thermo Scientific, Waltham, MA), 1× penicillin-streptomycin (Life Technologies), and 1× glutamax (Life Technologies). Cells were maintained in a 5 % CO_2_ atmosphere at 37°C and harvested at day 3 after plating. Neutralizing netrin-1 antibody 2F5 and the isotypic control H4 were obtained from Netris Pharma (Lyon, France). ER stress was induced by treating cells with tunicamycin (Tu) (Sigma-Aldrich, St. Louis, MO) or dithiothreitol (DTT) (Sigma-Aldrich) as indicated before harvest.

### Mice Models

All trials were performed under Institutional Review Board agreement CECCAP_CLB_2014_015. Six-week-old C57BL6 mice (Charles River Laboratory, Saint-Germain-Nuelles, France) were treated intraperitoneally with 1 mg/kg Tu or phosphate-buffered saline (PBS) for 24 hours and killed. Rosa-Lox-Stop-Lox (LSL)-netrin-1 transgenic mice conditionally overexpress flag-tagged netrin-1 under the control of a Rosa26 promoter. These animals were crossed with Rosa-CreERT2 (tamoxifen-dependent Cre recombinase) +/+ mice to generate breeder pairs of control and conditional overexpressers. Each mouse carries one copy of the CreERT2 transgene and was genotyped for LSL–netrin-1. At the age of 8 weeks, mice were injected intraperitoneally with 100 μL of 10 mg/mL tamoxifen (diluted in corn oil/ethanol, 9/1) daily, for 3 consecutive days to induce netrin-1 overexpression. After 2 weeks, mice were genotyped and netrin-1–overexpressing mice and their breeder pairs of control were treated with Tu or PBS for UPR induction for 24 hours and then killed.

### Quantitative and Conventional Reverse-Transcription Polymerase Chain Reaction

For quantitative reverse-transcription polymerase chain reaction (qRT-PCR), total RNA was extracted from cultured cells using the Extract-all reagent (Eurobio, Courtaboeuf, France) or the Nucleospin RNA/protein kit (Macherey-Nagel, Duren, Germany) for liver samples. RNA (1 μg) was treated with DNase I (Promega, Madison, WI), and then reverse transcribed in the presence of 5% dimethyl sulfoxide, using the Moloney Murine Leukemia Virus Reverse Transcriptase enzyme, according to the manufacturer’s instructions (Invitrogen). Real-time qRT-PCR was performed on a LightCycler 480 device (Roche, Basel, Switzerland) using the iQTM SYBR 533 Green Supermix (Bio-Rad, Hercules, CA). Dimethyl sulfoxide (10%; Sigma-Aldrich) was added to the PCR reaction for human netrin-1 quantification. Conventional RT-PCR was performed to amplify unspliced and spliced forms of XBP1 messenger RNA (mRNA), using the GoTaq DNA Polymerase according to the manufacturer’s instructions (Promega). XBP1 RT-PCRs were loaded on a 4% agarose gel to allow the separation between the 2 isoforms. PCR primer sequences and conditions are listed in Supplementary Tables 1 and 2.

### Isolation of Polysomal RNAs

Isolation of polysomal RNAs was performed as described previously.^34^ RNA was extracted using acid phenol and RNA integrity was monitored by gel electrophoresis on a 1.2% agarose gel. Densitometry (GelDoc; Bio-Rad) was used to determine fractions in which the 28S/18S ratio equaled 1.6 (ie, fractions corresponding to polysome-bound RNA). Specific mRNA distribution in the sucrose gradient was determined by qRT-PCR as described in the previous section with an equal volume of RNA from each fraction.

### Bicistronic Approach: Cloning Strategy

Bicistronic constructs were generated based on the Bicistronic plasmid (pBic) vector described by Giraud et al.^35^ Sequences corresponding to the HCV IRES (nucleotides 1–376) or the netrin-1 5’ untranslated region (UTR) (nucleotides 1–107) were synthesized by Genscript (Hong Kong, China) and then subcloned into the pBic vector between the Renilla and Firefly Luciferase coding regions after digestion with *EcoRI* and *NarI*. The cytomegalovirus (CMV) promoter was deleted from the pBic netrin-1 5’UTR after digestion by HindIII and BglII to obtain the pBic netrin-1 5’UTR ΔCMV promoter.

### DNA Templates and RNA Synthesis

To monitor dimethyl sulfate (DMS) and N-methyl-isatoic anhydride (NMIA) reactivity of the netrin-1 5’UTR, a 45-nucleotide cassette was attached to its 3’ terminus, as previously described.^36^ This cassette has been reported to fold autonomously into 2 short, stable hairpins^37^ that do not interfere with the predicted folding of the 5’UTR of netrin-1. It also contains a primer binding site for efficient complementary DNA synthesis. Briefly, the DNA template (T7p-5’UTR_ netrin-1*)* was obtained from the plasmid pGL-netrin-1 5’UTR 1–294 by amplification using the oligonucleotides T7p5’UTR_ netrin-1 and cas-as5’UTR_ netrin-1 (Supplementary Table 3). The RNA encoding the HCV IRES, 5’HCV-698, was obtained after in vitro transcription of *Bam*H1 digested pU5’HCV-691 plasmid.^38^ RNA100 was obtained by in vitro transcription from the plasmid pBSSK (Promega) previously digested with *Xba*I. Internal radiolabeling of RNA transcripts used for the 40S binding assays was essentially performed as reported.^39^ RNA synthesis was performed using the TranscriptAid T7 High Yield Transcription Kit following the manufacturer’s instructions (Thermo Scientific). The resulting transcripts were purified as previously described.^40^

### 40S Ribosomal Subunit Purification

Ribosomal particles (40S) were isolated from Huh-7 cell S10 extracts essentially as described.^41^ Briefly, Huh-7 cells were grown to 90% confluence in 10% calf serum–supplemented Dulbecco's modified Eagle medium, washed twice with cold PBS, treated with trypsin, and collected by centrifugation. Pellets were washed twice with 10 volumes of isotonic buffer (35 mmol/L Hepes-KOH pH 7.6, 146 mmol/L NaCl, and 11 mmol/L glucose) and diluted further into 1.5 volumes of hypotonic solution (20 mmol/L Hepes-KOH, pH 7.6, 10 mmol/L KCl, 1.5 mmol/L magnesium acetate, 1 mmol/L DTT, and protease inhibitors). The solution was incubated for 20 minutes at 4°C, supplemented with 0.2 volume of S10 buffer (100 mmol/L Hepes-KOH, pH 7.6, 600 mmol/L AcK, 20 mmol/L magnesium acetate, 25 mmol/L DTT, and protease inhibitors), and then broken with 20 strokes of a glass dounce homogenizer. A postnuclei supernatant was obtained by centrifugation at 5000*g* for 10 minutes. Polysomes were precipitated from this lysate by ultracentrifugation at 40,000 rpm (70.1 Ti rotor; Beckman, Brea, CA) for 4 hours in a 0.25 mol/L sucrose solution containing 20 mmol/L Tris-HCl, pH 7.6, 2 mmol/L DTT, 6 mmol/L MgCl_2_, and 0.5 mol/L KCl (buffer A). Pellets were diluted in buffer B (20 mmol/L Tris-HCl, pH 7.6, 2 mmol/L DTT, 6 mmol/L MgCl_2_, and 150 mmol/L KCl) to a concentration of 50–150 *A*260 U/mL. This suspension was incubated with 4 mmol/L puromycin for 10 minutes at 4°C and for 30 minutes at 37°C before the addition of KCl to a final concentration of 0.5 mol/L. Ribosomal subunits were resolved by centrifugation of 0.3-mL aliquots of this suspension through a 10%–30% sucrose gradient in buffer A for 17 hours at 4°C and 28,000 rpm, using a Beckman SW40 rotor. Subunits (40S) were concentrated from 0.5-mL gradient fractions with Amicon Ultracel-10k (Millipore Billerica, MA).

### Assembly of Netrin-1 5’UTR–40S Complexes and Filter Binding Assays

To generate RNA–40S subunit complexes, ^32^P end-labeled 5’UTR netrin-1 RNA constructs were first denatured by heating at 95°C for 2 minutes and then cooled to 4°C. Binding reactions were initiated by mixing 1 nmol/L of the RNA transcript in folding/binding buffer (30 mmol/L Hepes-NaOH, pH 7.4, 100 mmol/L sodium acetate, 5 mmol/L magnesium acetate, and 2 mmol/L DTT) with increasing concentrations of the 40S ribosomal subunit. Reactions were incubated at 37°C for 30 minutes before loading on 0.45-μm nitrocellulose filters (GE Healthcare, Little Chalfont, UK). The filters were presoaked in the binding buffer, assembled in a dot blot apparatus, and the samples then were added directly onto the filter under vacuum. The filters then were removed, dried, and scanned in a Phosphorimager (Storm 820; GE Healthcare) and quantified with Image Quant 5.2 software (GE Healthcare). Values are the average of at least 3 independent experiments. For the competition reactions, 40S-5’UTR assembly was performed as described earlier in the presence of a molar excess of the unlabeled transcripts RNA100, 5’UTR netrin-1, or 5’HCV-698 HCV IRES.

### DMS Probing

DMS chemical probing was performed essentially as previously described.^40^ Fluorescently labeled DNA oligonucleotides (Applied Biosystems, Carlsbad, CA) used for primer extension reactions were purified using high-resolution denaturing polyacrylamide gels. Primer Std (Supplementary Table 3), which anneals within the structure cassette inserted at the 3’ end of the respective transcript, was labeled fluorescently with NED (to detect untreated and treated probes) or VIC (for sequencing reaction). For the primer extension reaction, 0.4 pmol of gel-purified primer were hybridized with the total processed RNA by incubation at 95°C for 2 minutes, followed by fast cooling at 4°C for 5 minutes and subsequent incubation at 52°C, to allow efficient annealing. Extension reactions were performed for 30 minutes at 52°C in a 20-μL reaction containing reverse-transcriptase buffer, 0.5 mmol/L deoxynucleoside triphosphate, and 100 U SuperScript III RT (Invitrogen). RNA sequencing reactions were performed under identical conditions with the VIC fluorescently labeled primer in the presence of 0.25 mmol/L of ddCTP 2′,3′-Dideoxycytidine 5′-Triphosphate. The resulting complementary DNA samples were purified and resolved as reported^40^, ^42^ in the genomic unit of the IPBLN-CSIC. Electropherograms were analyzed using QuSHAPE software.^43^ Normalized DMS reactivity values for each nucleotide position were obtained by dividing each value by the average intensity of the 10% most reactive residues, after excluding outliers calculated by box plot analysis.

### Selective 2’-Hydroxyl Acylation and Primer Extension Analysis

Selective 2’-hydroxyl acylation and primer extension analyses were performed by treatment with NMIA as previously described.^40^ Normalized NMIA reactivity values for each nucleotide position were calculated as indicated for DMS probing.

### Secondary Structure Prediction

RNA secondary structure models were generated using ShapeKnots software (University of Rochester, Rochester, NY),^44^ including the structural constraints derived from NMIA and DMS relative reactivity data.

### Small Interfering RNA–Mediated Knockdown

A total of 2 × 10^4^ cells/cm^2^ were transfected with 25 nmol/L final concentration of a nontargeting control small interfering RNA (siRNA), netrin-1 siRNA, UNC5A siRNA, UNC5C siRNA, DAPK1 siRNA, or PR65β siRNA (designed by Sigma-Aldrich) using Lipofectamine 2000 (Invitrogen) according to the manufacturer’s instructions. Sequences of siRNAs are listed in Supplementary Table 4.

### Plasmid Transfection

A total of 3 × 10^5^ cells were transfected with 2.5 μg netrin-1 or neuronal vanilloid receptor 1 (VR1) expression plasmids or bicistronic constructs using Lipofectamine 2000 (Invitrogen), according to the manufacturer’s instructions.

### Caspase-3 and Proliferation Assays

Caspase-3 activity assays were performed using the caspase 3/CPP32 Colorimetric Assay Kit, according to the manufacturer’s instructions (Gentaur Biovision, Kampenhout, Belgium). The cell proliferation assay was performed using neutral red uptake standard assay.^45^

### PP2A Activity Assays

PP2A activity was measured using the active PP2A DuoSet IC kit according to the manufacturer’s instructions (R*&D Systems,* Minneapolis, MN*).*

### Fluorescence-Activated Cell Sorter Analyses

HepaRG cells were detached with Versene buffer (Life Technologies), washed twice in PBS, and centrifuged at 1200 rpm for 5 minutes. Cells then were stained in a mix containing 1 mg/mL RNase-A (Invitrogen) and 100 μg/mL propidium iodide (Sigma-Aldrich) for 5 minutes at room temperature, washed in PBS, resuspended in PBS supplemented with 10 mmol/L EDTA, and analyzed using a FACscalibur device (BD, Franklin Lakes, NJ).

### Immunoblotting

Immunoblotting was performed using standard protocols with antibodies against the human influenza hemagglutinin (HA) tag (Sigma-Aldrich), FLAG-M2 (Sigma-Aldrich), β-actin (Sigma-Aldrich), total DAPK1 (Sigma-Aldrich), PR65β (Abcam, Cambridge, UK), netrin-1 antibody (AF1109; R&D Systems), eIF2α (Cell Signaling), Phospho-eIF2α (9721, Ser-51 specific; Cell Signaling), and α-tubulin (Thermo Scientific). Antibody information is available in Supplementary Table 5.

### Terminal Deoxynucleotidyl Transferase–Mediated Deoxyuridine Triphosphate Nick-End Labeling Staining and Netrin-1 Immunochemistry

Terminal deoxynucleotidyl transferase–mediated deoxyuridine triphosphate nick-end labeling (TUNEL) staining and netrin-1 immunochemistry were performed by the Anipath core facility (Inserm, Lyon, France), using the R&D systems TUNEL kit and an antibody directed against netrin-1 (MAB1109; R&D Systems) (Supplementary Table 5).

### Northern Blot

Cells transfected with the previously mentioned constructs were lysed in TRIzol (Life Technologies). A total of 10 μg of total RNA extracts were denatured in glyoxal and underwent agarose gel electrophoresis, transferred onto a nylon membrane by capillary blotting, blocked, and hybridized using the Church and Gilbert procedure with 20 pmol of Fluc reverse primer (Supplementary Table 3) previously labeled with ^32^P.

### Statistical Analysis

Statistical analysis was conducted using the Mann–Whitney or Wilcoxon tests with GraphPad Prism (La Jolla, CA) software 5.0. Significance was as follows: *P < .05, **P < .01, ***P < .001.

## Results

### The UPR Is Functional in HepaRG Cells

To analyze the role of netrin-1 in the UPR in the liver, we first verified the presence of a functional UPR in HepaRG cells. HepaRG cells are a recognized, untransformed, human liver cell line that closely resembles primary human hepatocytes.^46^, ^47^ We treated cells with different doses of DTT or Tu and monitored translational status by evaluation of the phosphorylation level of eIF2α at Ser-51, a mark associated with a rapid decrease in protein biosynthesis.^48^ We also monitored the transcriptional induction of a number of UPR-related genes over time. These included *GADD34*, total *XBP1*, and *p58*^*IPK*^, which are transcriptional targets of the activated PERK, ATF6, and IRE1α pathways, respectively,^13^ and *GRP94* and *CHOP* mRNA levels, up-regulated during UPR. In addition, *XBP1* mRNA splicing, another hallmark of IRE1α pathway activation,^13^ also was monitored.

**Figure 1:**
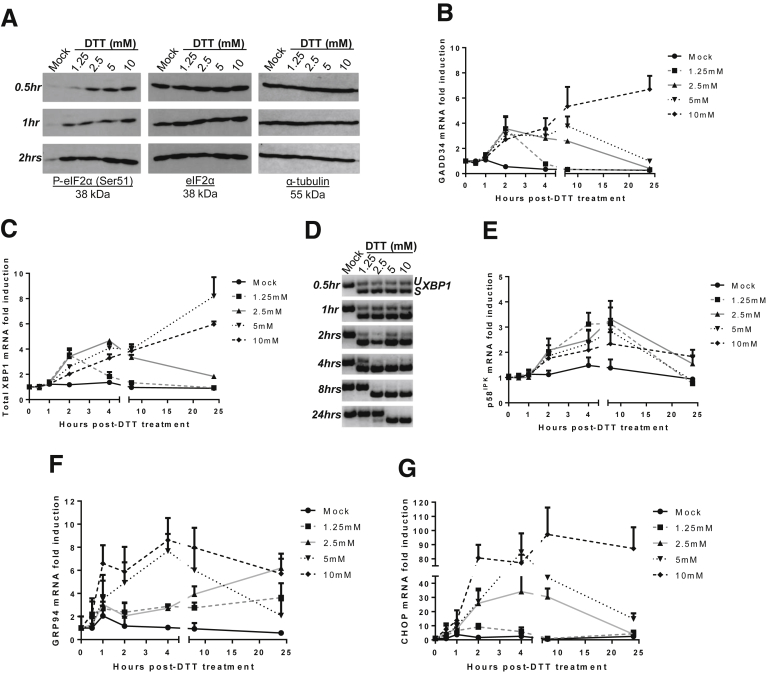
**The UPR is induced upon DTT treatment in HepaRG cells.** After DTT treatment, protein and total RNA were extracted and subjected to Western blot, qRT-PCR, or RT-PCR. (*A*) Immunoblotting showing eIF2α phosphorylation levels. Results were identical from 2 to 24 hours after treatment. Representative experiment of n = 3. (*B*) Quantification of *GADD34* mRNA (dependent on the PERK pathway) (mean + SEM, n = 3). (*C*) Quantification of Total *XBP1* mRNA (dependent on the ATF6 pathway) (mean + SEM, n = 3). (*D*) RT-PCR for detection of *uXBP1* (unspliced isoform) and *sXBP1* (spliced isoform) (dependent on the IRE1α pathway) on agarose gel. Representative results, n = 3. No qPCR signal could be obtained using samples retrotranscribed with heat-inactivated RT. (*E–G*) Quantification of *p58*^*IPK*^ mRNA (dependent on the IRE1α pathway), *CHOP* mRNA, and *GRP94* mRNA (mean + SEM, n = 3). See also Supplementary Figure 1.

**Figure 2:**
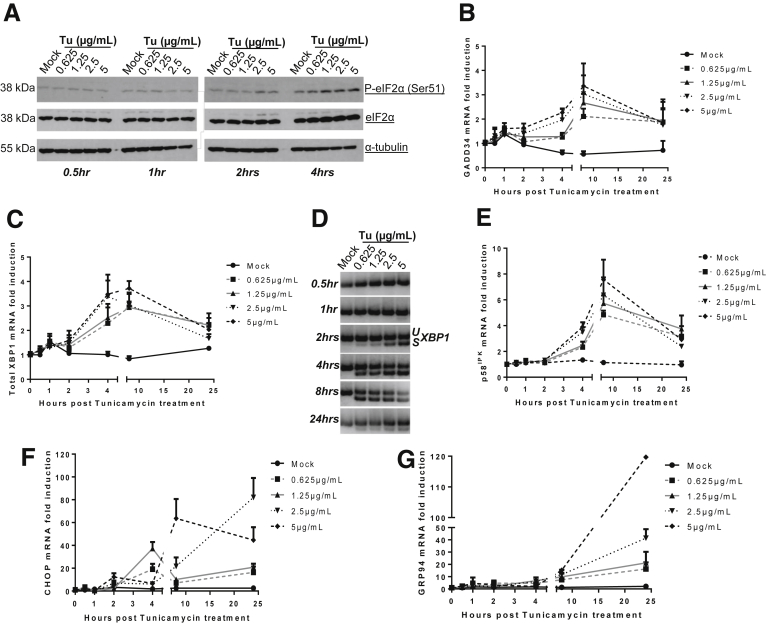
**UPR is induced upon tunicamycin treatment in HepaRG cells.** After tunicamycin treatment, protein and total RNA were extracted in a time- and dose-dependent manner and subjected to Western blot, qRT-PCR, or RT-PCR. (*A*) Immunoblotting showing eIF2α phosphorylation level at Ser-51 (P-eIF2α [Ser-51]). Results were identical from 4 to 24 hours after treatment. Representative experiment of n = 3. (*B*) Quantification of *GADD34* mRNA (dependent on the PERK pathway) (mean + SEM, n = 3). (*C*) Quantification of Total *XBP1* mRNA (dependent on the ATF6 pathway) (mean + SEM, n = 3). (*D*) RT-PCR for detection of *uXBP1* (unspliced isoform) and *sXBP1* (spliced isoform) (dependent on the IRE1α pathway) on agarose gel. Representative results, n = 3. No qPCR signal could be obtained using samples retrotranscribed with heat-inactivated RT. (*E–G*) Quantification of *p58*^*IPK*^ mRNA (dependent on the IRE1α pathway), *CHOP* mRNA, and *GRP94* mRNA (mean + SEM, n = 3). See also Supplementary Figure 1.

As expected, eIF2α phosphorylation level at Ser-51 was increased in a time- and dose-dependent manner after DTT treatment, indicating protein translation shutdown. At 2 hours after treatment, eIF2α was completely phosphorylated at Ser-51 (Figure 1*A*). From 4 to 8 hours after adding DTT, *GADD34* and total *XBP1* mRNA levels were up-regulated by 4- to 6-fold, respectively (Figure 1*B* and 1*C*). Splicing of *XBP1* mRNA was detected as early as 30 minutes after treatment with DTT, increased in a time- and dose-dependent manner, and reached its maximum at 4 hours after treatment with 2.5 mmol/L DTT (Figure 1*D*). *p58*^*IPK*^ mRNA levels also were induced by approximately 3-fold at 4–8 hours after treatment (Figure 1*E*). *GRP94* and *CHOP* mRNA levels also increased up to 10- and 100-fold at 4–8 hours after treatment (Figure 1*F* and 1*G*). UPR-related transcripts also were induced, yet substantially later and weaker after having treated HepaRG cells with Tu (Figure 2). Altogether, these results indicate that the UPR is functional in HepaRG cells. To generate robust and reproducible UPR induction, DTT was chosen for all subsequent in vitro experiments and *XBP1* mRNA splicing served as an indicator for a functional UPR.^49^

### Netrin-1 Translation Is Resistant to UPR-Related Translational Shutdown in Human Cells

**Supplementary Figure 1:**
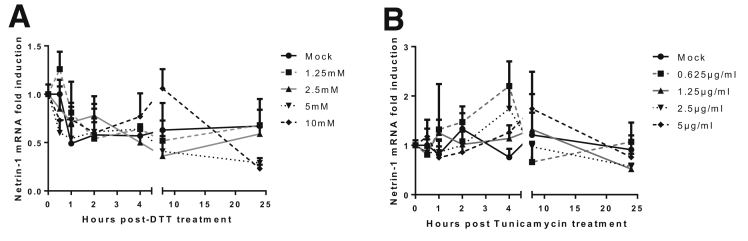
***Netrin-1* mRNA level is not modulated during UPR.** HepaRG cells were treated with (*A*) DTT or (*B*) tunicamycin and subjected to RT-qPCR for quantification of netrin-1 mRNA (Mean + sem, n = 3).

**Supplementary Figure 2:**
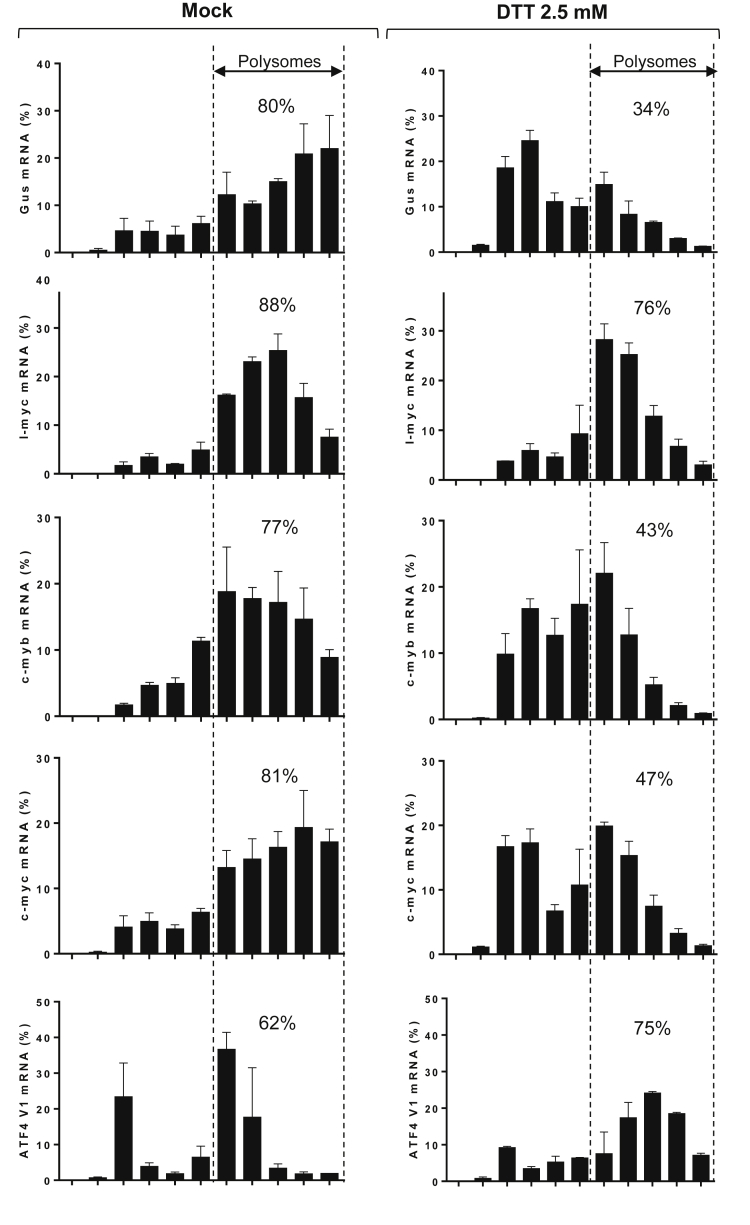
**Differential association of Netrin-1 5’UTR-related transcripts with translational units during UPR in HepaRG cells.** HepaRG cells were treated with 2.5 mM DTT for 4 hours or left untreated (Mock). Cell lysates were subjected to polysomes fractionation followed by RT-qPCR for quantification of gus, l-myc, c-myb, c-myc and ATF4 V1 mRNAs. Bar graphs depict Gus, l-myc, c-myb, c-myc and ATF4 V1 mRNA quantification across fractions.

**Supplementary Figure 3:**
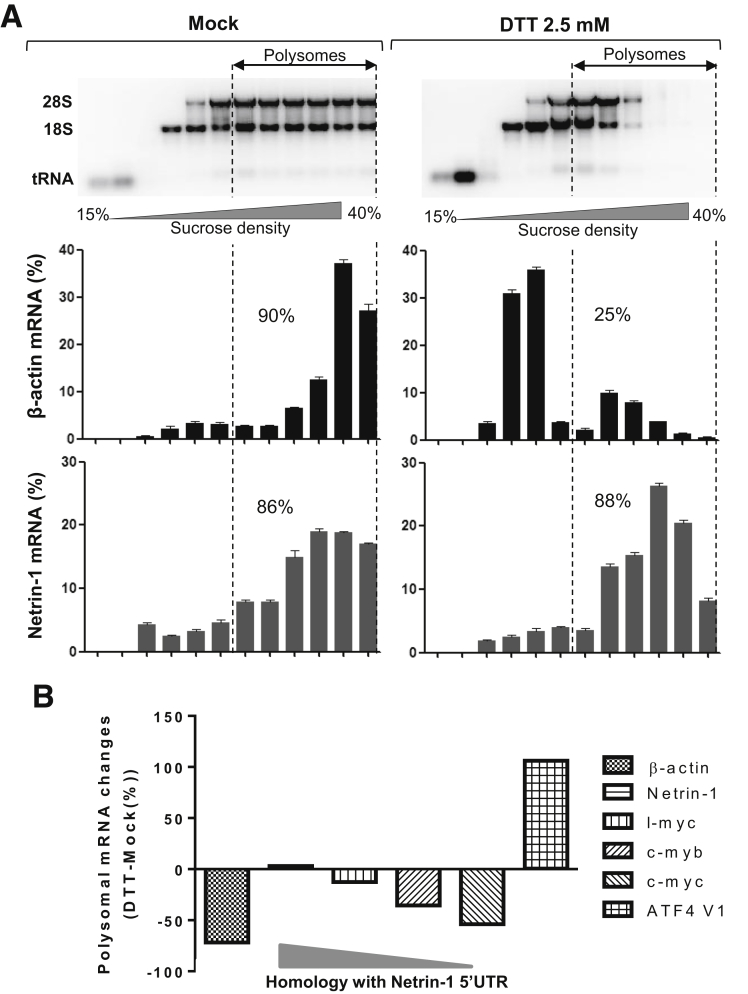
**Netrin-1 mRNA association with translational units is unchanged during UPR in Huh7.5 cells.** Huh7.5 cells were treated with 2.5 mM DTT for 4 hours of left untreated (Mock). Cell lysates were subjected to sucrose gradient fractionation followed by RT-qPCR for quantification of β-actin, netrin-1, l-myb, c-myb c-myc and ATF4 V1 mRNA. (*A*) Distribution of mRNAs in the sucrose gradient. Agarose gel electrophoreses show ribosomal RNA distribution across sucrose gradient. Bar graph represents RNA fractions processed for β-actin and netrin-1 mRNA quantification. (Mean + sem). (*B*) The evolution of the association of indicated mRNAs with polysomes was determined as percentage difference of polysome-associated mRNA signals in DTT samples vs mock samples in each polysome profile.

**Supplementary Figure 4:**
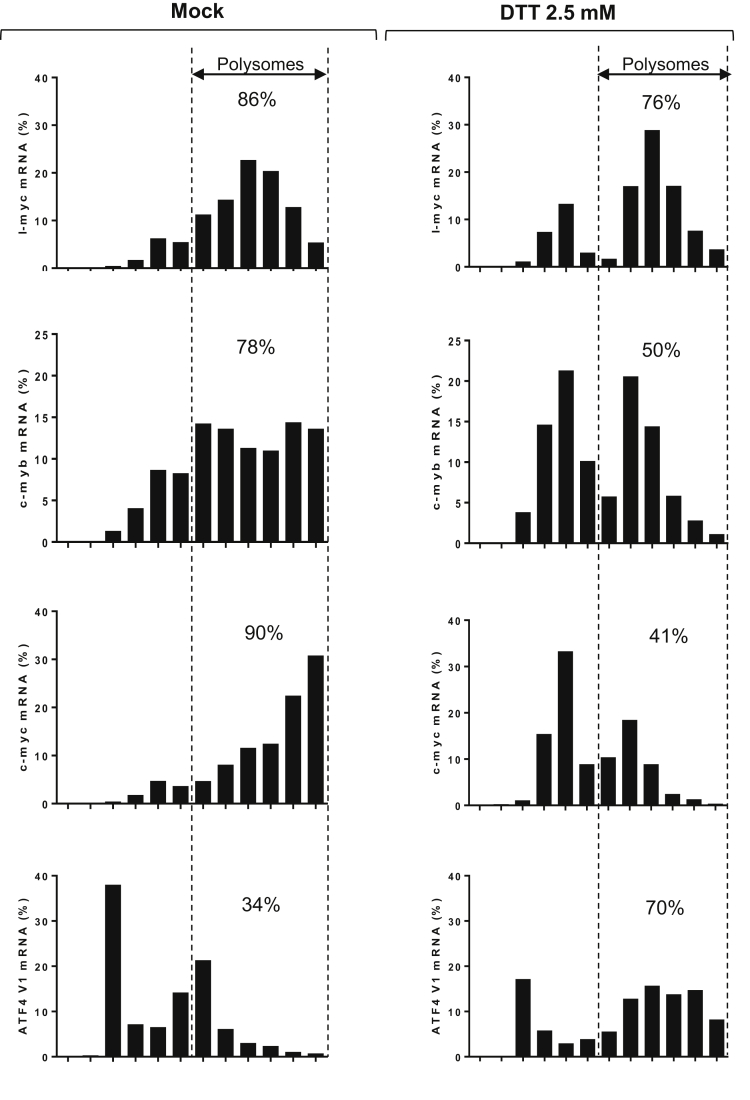
**Differential association of Netrin-1 5’UTR-related transcripts with translational units during UPR in Huh7.5 cells.** Huh7.5 cells were treated with 2.5 mM DTT for 4 hours of left untreated (Mock). Cell lysates were subjected to sucrose gradient fractionation followed by RT-qPCR for quantification of l-myb, c-myb c-myc and ATF4 mRNA. Figure shows distribution of mRNAs across sucrose gradients.

**Figure 3:**
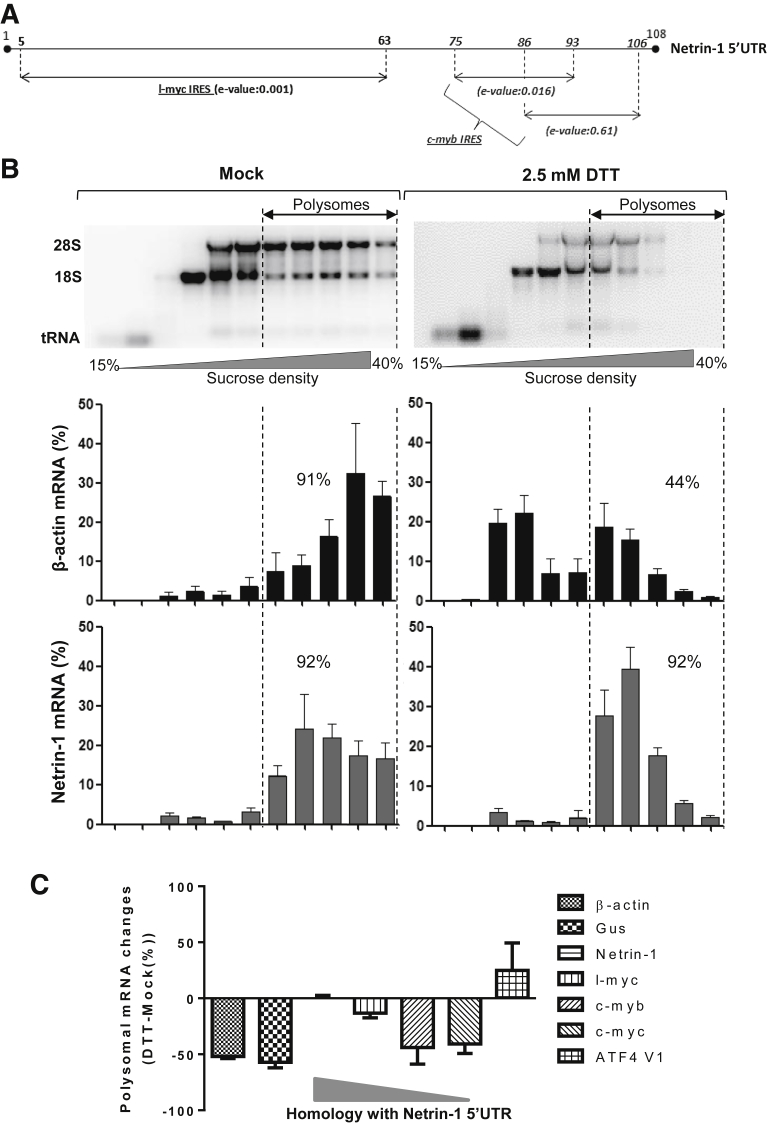
**Netrin-1 mRNA association with translational units is resistant to the UPR.** HepaRG cells were treated with DTT for 4 hours. Cell lysates were subjected to polysome fractionation followed by qRT-PCR for quantification of *GUS*, *β-actin*, *netrin-1*, *l-myc*, *c-myb, c-myc*, and *ATF4* V1 mRNAs. (*A*) *netrin-1* 5’UTR is similar to l-myc (e-value, 0.001) and c-myb (e-values, 0.016 and 0.61). Bioinformatic analysis was performed using IRESite (http://iresite.org). (*B*) Distribution of mRNAs after sucrose gradient fractionation. *Bar graphs* represent *β-actin* and *netrin-1* mRNA quantification. Agarose gel electrophoreses represent ribosomal RNA distribution across gradient. (*C*) The evolution of the association of indicated mRNAs with polysomes upon DTT treatment was determined as the percentage difference of the polysome-associated signals in DDT vs mock samples in each profile (mean + SEM, n = 3). See also Supplementary Figure 2, Supplementary Figure 3, Supplementary Figure 4. tRNA, transfer RNA.

Next we assessed if the UPR affected netrin-1 expression in the samples from Figure 1. Interestingly, total netrin-1 mRNA levels were insensitive to the UPR (Supplementary Figure 1). Because the UPR has a strong effect on translation, we further investigated the effect of the UPR on netrin-1 translation by quantifying the association of netrin-1 mRNA with polysomes through sucrose gradient fractionation. Polysome association of *β-actin* and *GUS* mRNAs served as control. In addition, IRES-bearing mRNAs encoding the oncogenes l-myc,^50^ c-myb,^51^ and c-myc^52^ were included. The latter were chosen based on the results from a basic local alignment tool search performed using the IRESite database, which suggested that the 5’UTRs of netrin-1, l-myc (e-value: 0.001) and c-myb (e-value: 0.016 and 0.61, depending on the region studied) are located between nucleotides 5 to 63 and 75 to 106, respectively (Figure 3*A*).^53^ c-myc was included as a control of an oncogenic transcript with an unrelated 5’UTR structure. To this end, HepaRG cells were treated with 2.5 mmol/L DTT for 4 hours, a setting in which UPR was activated efficiently. As expected, polyribosomal dissociation was observed in response to DTT (Figure 3*B*). The position of the earlier-mentioned mRNAs across the sucrose gradient were measured by qRT-PCR. The limit between nonpolysomal and polysomal fractions was set based on the 28S/18S intensity ratio. In this setting, *β-actin* mRNA association with polysomes decreased from 91% to 44% after DTT treatment (corresponding to a reduction of 50%), whereas *netrin-1* mRNA association with polysomes remained unchanged (92%) (Figure 3*B*). Similar results ranging from 15% to more than 40% changes were observed for gus, l-myc, c-myb, and c-myc (Supplementary Figure 2). In contrast, polysome association of the IRES-bearing *ATF4* transcript variant 1 mRNA (*ATF4 V1*)^54^ (not indexed in the IRESite database), which is known to be induced during UPR, was increased by approximately 30%. Interestingly, polysome association of *l-myc*, *c-myb*, or *c-myc* transcripts decreased with their decreasing similarity with the *netrin-1* 5’UTR (Figure 3*C*). Similar results were obtained in Huh7.5 cells, a human hepatocytic cell line (Supplementary Figures 3 and 4). Altogether, our data suggest that *netrin-1* mRNA remains associated with translational units during the UPR, suggesting its involvement in UPR-associated processes mediating cell survival.

### Netrin-1 mRNA Is Translated Through IRES-Dependent Translation

**Figure 4:**
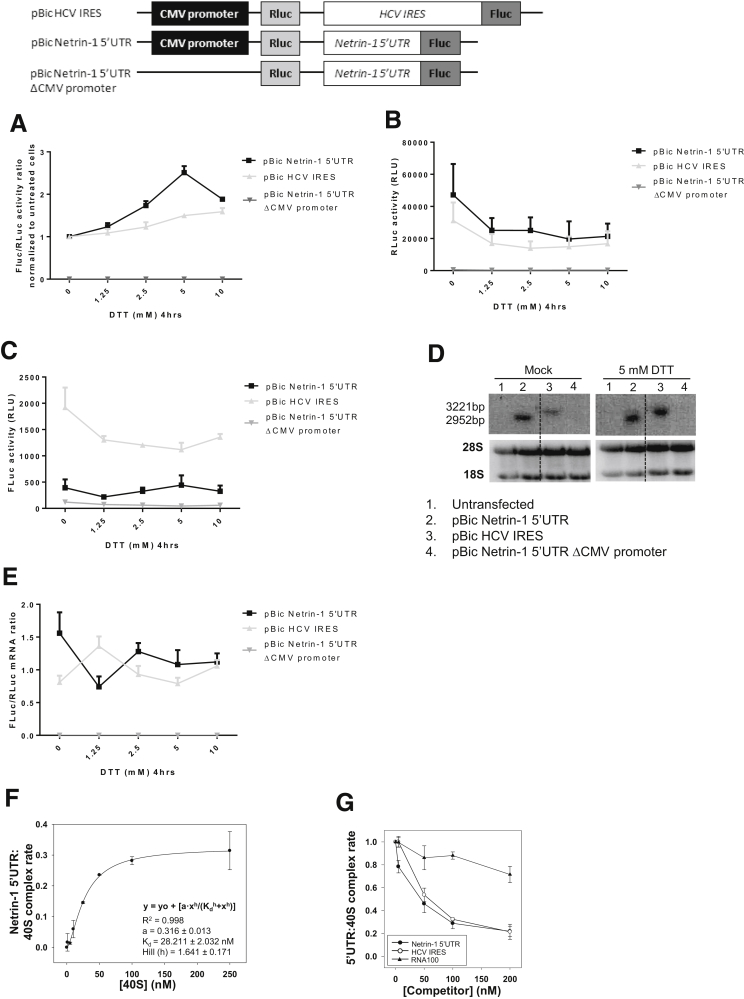
***Netrin-1* mRNA is translated through IRES-dependent translation.** HepaRG cells were transfected with bicistronic vectors carrying the *netrin-1* 5’UTR or the HCV IRES between the Rluc and Fluc coding regions. Three days after transfection, cells were treated for 4 hours with increasing amounts of DTT or left untreated. (*A*) The netrin-1 5’UTR allows Fluc translation in a bicistronic construct upon UPR. (*B*) Renilla luciferase activity (RLU) is decreased after DTT treatment. (*C*) Firefly luciferase activity (RLU) is differentially modulated after DTT treatment. (*D*) Northern blot showing the presence of a unique mRNA population of the expected size being synthesized. Equal loading was confirmed by *18S* and *28S* intensities. (*E*) FLuc/RLuc mRNA ratio is unchanged after DTT treatment. (*F*) The interaction between the *netrin-1* 5’UTR and the 40S ribosomal subunit is concentration-dependent. Internally ^32^P-labeled *netrin-1* 5’UTR transcript was incubated with purified 40S particles in binding buffer. Complexes were detected by filter retention (mean ± SD, n = 3). (*G*) The interaction between the *netrin-1* 5’UTR and *40S* subunit is displaced by the HCV IRES. The internally ^32^P-labeled *netrin-1* 5’UTR RNA was incubated with the purified 40S particle in the presence of increasing amounts of the unlabeled transcripts (mean ± SD, n = 3).

One possible explanation for the earlier-described findings would be the existence of an IRES element in the 5’UTR of the *netrin-1* mRNA. To test this hypothesis, bicistronic constructs containing the 5’UTR of *netrin-1* or the HCV IRES between the Renilla luciferase (*rluc*) and the Firefly luciferase (*fluc*) gene were generated.^35^ In these constructs, RLuc translation is initiated by a cap-dependent manner and FLuc synthesis depends on the potential ribosome recruitment mediated by the inserted netrin-1 5’UTR sequence. The Fluc/Rluc activity ratio increased in a dose-dependent manner after DTT treatment (Figure 4*A*). Indeed, cap-dependent translation measured as RLuc activity was decreased substantially, whereas FLuc synthesis, which is driven by the 5’UTR of *netrin-1*, was not affected (Figure 4*B* and *C*). This is in good concordance with the observation that the HCV IRES also was able to efficiently drive FLuc translation during the UPR but at a lower level than the netrin-1 5’UTR (Figure 4*A*). No luciferase activity was detected using vectors lacking the CMV promoter (Figure 4*B* and *C*). Moreover, a unique mRNA population was detected by Northern blot and FLuc/RLuc mRNA ratios were not modified after DTT treatment, indicating that the *netrin-1* 5’UTR sequence is devoid of promoter activity and of cryptic splice site (Figure 4*D* and E). The observation that the 5’UTR of the *netrin-1* mRNA can promote the translation of an internal cistron is consistent with the presence of an IRES. To further corroborate this hypothesis, we assessed recruitment of the 40S ribosome by the netrin-1 5’UTR. As shown in Figure 4*F*, the *netrin-1* 5’UTR recruits the 40S particle with a low nanomolar affinity range (K_d_ = 28.21 ± 2.03 nmol/L). No competition was observed using a nonrelated molecule, RNA 100, verifying the specificity of the interaction. Interestingly, an RNA transcript containing the HCV IRES^38^ efficiently displaced the interaction between the *netrin-1* 5’UTR and the 40S subunits to a similar extent as the nonlabeled *netrin-1* 5’UTR (Figure 4*G*). This suggests that the 5’UTR of *netrin-1* mRNA recruits the 40S ribosomal subunit in the absence of any other translation factor and with similar efficiency to the HCV IRES. Taken together, our results support the presence of an IRES in the 5’UTR of the *netrin-1* mRNA.

### Structural Mapping of the 5’UTR Netrin-1 mRNA

There is an intimate relationship between the function of an RNA molecule and its architecture. This prompted us to analyze the folding of the 5’-end of the netrin-1 mRNA, comprising the putative IRES and the first few nucleotides of the coding sequence. To this end, RNA was analyzed by DMS chemical probing assays and selective 2’-hydroxyl acylation and primer extension analyses with a NMIA reagent.

**Figure 5:**
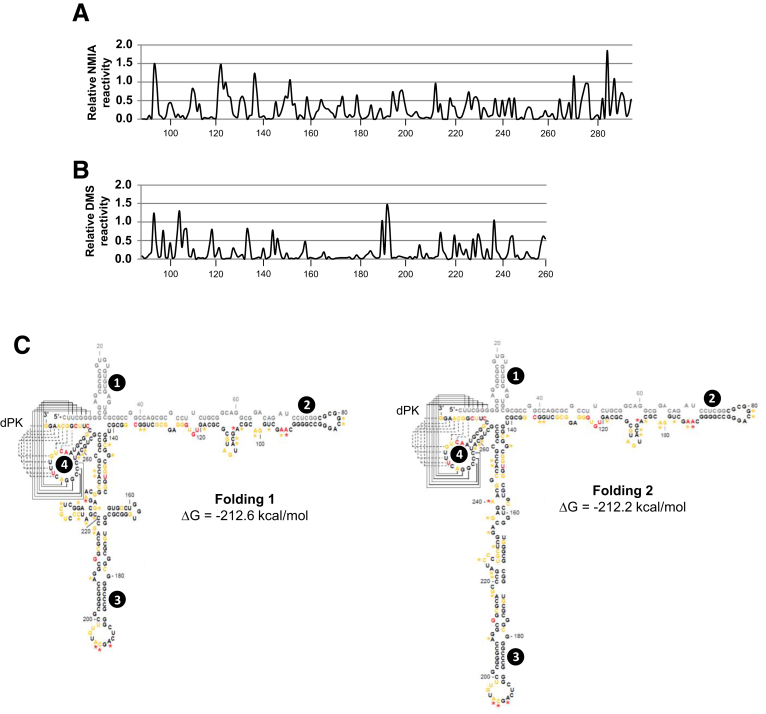
**Secondary structure models of the netrin-1 IRES.** (*A* and *B*) *Line graphs* show values of (*A*) NMIA and (*B*) DMS reactivities at each nucleotide position for the netrin-1 construct under study (mean + SEM, n = 3). (*C*) Secondary structure models as predicted by the software ShapeKnots using selective 2’-hydroxyl acylation and primer extension and DMS data. NMIA and DMS reactivity data are denoted on a black, yellow, and red scale for low, medium, and high SHAPE reactivities, respectively. dPK, double pseudoknot element. Major stem-loops (2 and 3); short hairpins (1 and 4). Numbers refer to nucleotide coordinates of netrin-1 (GenBank accession no: NM_004822.2).

Both DMS and NMIA reactivity profiles showed a low global mean reactivity value (Figure 5*A* and *B*), with local and well-delimited average reactivity peaks, suggesting a compact folding with long stem structures closed by apical loops. The experimental data then were used to further define secondary structure models using the ShapeKnots tool provided by the RNAStructure software package (Mathews lab software, Rochester, NY).^44^ As shown in Figure 5*C*, our analysis yielded a well-defined architecture with 2 major stem loops (regions 2 and 3), flanked by 2 short hairpins (1 and 4). Furthermore, it provided an interesting view of the 3’ end of the 5’UTR, suggesting this specific region showed a relatively relaxed folding with 2 possible conformations (folding 1 and 2). Both architectures can be considered thermodynamically equivalent structural isoforms, likely being transitions from one to another. In the first conformation, stem 3 is organized around a 3-way junction, which may serve as a protein recruiting platform.^55^ In the second version, the RNA adopts a structure resembling a single long stem interrupted by internal loops and bulges, mainly defined by noncanonical RNA G-A base pairs, which frequently are found in these so-called E-loops. Interestingly, a common and unique double pseudoknot element formed by the very 5’-end of the 5’UTR of the sequence was preserved in both conformations.

Although complete experimental evidence for these structures remains to be generated, these structural data reflect the probable existence of 2 prominent structural isoforms, which could be related to a translational switch mechanism, from cap-dependent to IRES-dependent translation.

### Netrin-1 Confers Protection Against UPR-Induced Cell Death

**Figure 6:**
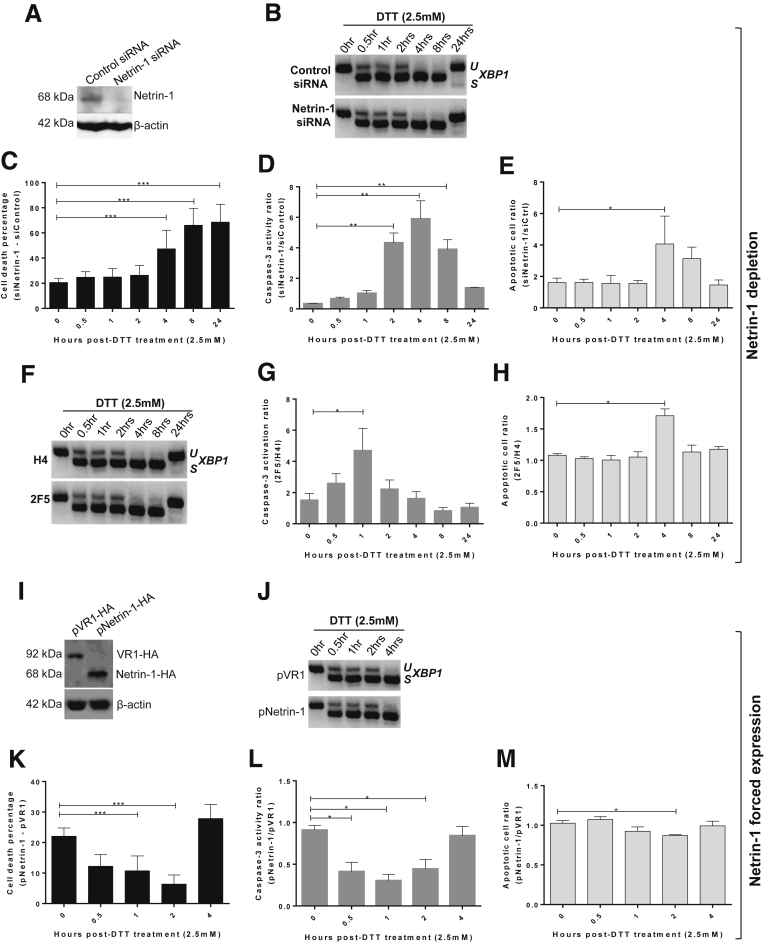
**Netrin-1 protects against cell death during UPR.** (*A–H*) Netrin-1 depletion. (*A–E*) HepaRG cells were transfected with netrin-1 siRNA, treated with DTT, and harvested in a time course assay. (*A*) Netrin-1 protein knockdown by siRNA was validated by immunoblot (representative result, n = 3). (*B*) *XBP1* splicing was confirmed by RT-PCR (representative result, n = 3). (*C*) UPR increases the dependence of cells toward netrin-1 for survival. *Graphs* indicate the difference in cell death (in percentages) (mean + SEM; n = 3; Mann–Whitney test; *P* < .05). (*D*) UPR increases the sensitivity of cells toward netrin-1 for caspase-3 activation. *Graphs* indicate caspase-3 activity ratios (mean + SEM; n = 3; Mann–Whitney test; *P* < .05). (*E*) Netrin-1 depletion increases apoptosis. *Graph* indicates apoptotic cell death ratio by propidium iodide staining and flow cytometry (mean + SEM; n = 3; Mann–Whitney test; *P* < .05). (*F–H*) Netrin-1 inhibition using a neutralizing antibody. HepaRG cells were seeded and treated with a control (H4) or anti–netrin-1 antibody (2F5) the same day. Cells were treated with DTT 3 days after addition and harvested after the indicated time points. (*F*) Assessment of XBP1 mRNA splicing (representative result, n = 3). (*G*) Netrin-1 neutralization enhances caspase-3 activity. *Graph* indicates the caspase-3 activity ratios between 2F5 and H4-treated cells (mean + SEM; n = 3; Mann–Whitney test; *P* < .05). (*H*) Netrin-1 depletion increases apoptosis. *Graph* indicates the apoptotic cell death ratio as assessed after propidium iodide staining and flow cytometry of netrin-1–depleted and control cells (mean + SEM; n = 3; Mann–Whitney test; *P* < .05). (*I–M*) Netrin-1 forced expression. HepaRG cells were transfected with control (VR1-HA) and netrin-1 (netrin-1–HA) vectors, treated with DTT, and harvested. (*I*) Netrin-1 expression was assessed by immunoblotting. Representative result, n = 3. (*J*) XBP1 mRNA splicing was quantified by RT-PCR at the indicated time points. Representative result, n = 3. (*K*) UPR increases the sensitivity of cells toward netrin-1 for protection against cell death in time course assays. *Graph* indicates the difference in cell death (in percentages) between netrin-1–overexpressing cells and control cells (mean + SEM; n = 3; Mann–Whitney test; *P* < .05). (*L*) UPR increases the sensitivity of cells toward netrin-1 for caspase-3 activation in time course assays. *Graph* indicates the ratios of caspase-3 activation levels among netrin-1–overexpressing and control cells (mean + SEM; n = 3; Mann–Whitney test; *P* < .05). (*M*) Netrin-1 overexpression decreases apoptosis in a time course assay. *Graph* indicates the apoptotic cell death ratio as assessed after propidium iodide staining and flow cytometry of netrin-1–overexpressing and control cells (mean + SEM; n = 3; Mann–Whitney test; *P* < .05). *,**, or *** refer to statistical analyses.

To determine whether translated netrin-1 indeed mediates cell survival during the UPR, we assessed the effect of netrin-1 depletion on several cell death parameters. First, HepaRG cells were transfected with control or netrin-1–targeting siRNAs and then treated with DTT over time. The netrin-1 protein level was reduced significantly, indicating a successful knockdown (Figure 6*A*). The *XBP1* mRNA profile showed an induction of the UPR (Figure 6*B*). Depletion of netrin-1 led to an increase in the percentage of dead cells (DTT vs mock) from 20% to 60% (Figure 6*C*), and a 6-fold enhancement of caspase-3 activity 4 hours after treatment (Figure 6*D*). In addition, apoptotic cells in the sub-G1 phase were determined by propidium iodide staining and flow cytometry. The number of apoptotic cells in netrin-1–depleted samples was increased up to 4-fold at 4 hours after treatment (Figure 6*E*). Comparable results were obtained using a netrin-1–neutralizing antibody instead of siRNA, confirming the protective role of netrin-1 and excluding off-target effects (Figure 6*F–H*).

To determine whether netrin-1 overexpression had the opposite effect, HepaRG cells were transfected with plasmids encoding either netrin-1–HA or VR1-HA as control and treated with DTT for up to 24 hours. Immunoblotting confirmed expression of netrin-1–HA and VR1-HA proteins in the transfected cells (Figure 6*I*). UPR induction was confirmed by consideration of *XBP1* splicing (Figure 6*J*). In contrast to netrin-1 knockdown, overexpression decreased cell death and caspase-3 activity by 2.5- to 3-fold (Figure 6*K* and *L*). Likewise, apoptotic cells were decreased by 20% (Figure 6*M*). Altogether, these results suggest that netrin-1 confers protection against UPR-induced cell death in hepatocytes in vitro.

### UNC5A/C Signaling Through DAPK1/PR65b Induces Caspase-3 Activation During UPR

**Figure 7:**
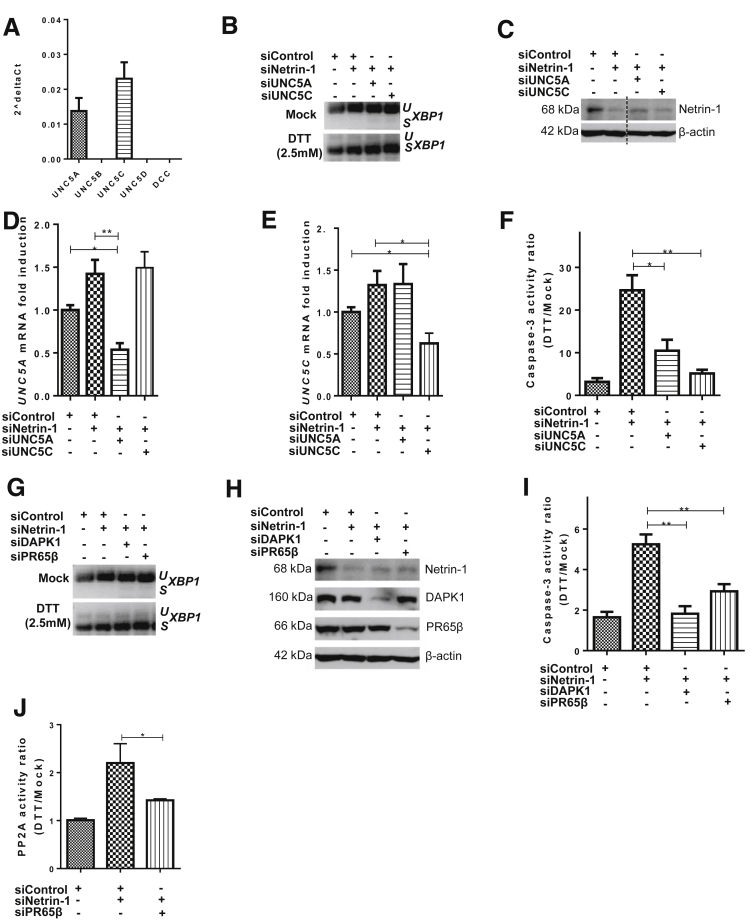
**UNC5A and UNC5C induce caspase-3 activation through DAPK1/PR65b during UPR.** (*A*) Quantification of netrin-1–receptor expression by qRT-PCR (mean + SEM, n = 3). (*B–F*) Identification of the implicated netrin-1 receptors. HepaRG cells were transfected with siRNAs and treated with DTT or not for 4 hours (mock). (*B*) Assessment of *XBP1* mRNA splicing by RT-PCR. Representative result, n = 3. (*C*) Assessment of netrin-1 protein knockdown by immunoblotting. Representative result, n = 3. (*D* and *E*) Assessment of transcript knockdown efficiencies. *Graphs* indicate (*D*) *UNC5A* and (*E*) *UNC5C* mRNA levels in siRNA-treated cells in comparison with control siRNA-treated cells (mean + SEM; n = 3; Mann–Whitney test; *P* < .05). (*F*) Caspase-3 activation is reversed by UNC5A or UNC5C knockdown after UPR induction. *Graph* indicates caspase-3 activity ratio of DTT vs untreated cells for each condition (mean + SEM; n = 3; Mann–Whitney test; *P* < .05). (*G–J*) Identification of the downstream signaling pathway. HepaRG cells were transfected with siRNAs and treated with DTT or not for 4 hours (mock). (*G*) Assessment of *XBP1* mRNA splicing by RT-PCR. Representative result, n = 3. (*H*) Evaluation of netrin-1, DAPK1, and PR65β depletion by immunoblotting. Representative result, n = 3. (*I*) Caspase-3 activation is reversed by DAPK1 or PR65β knockdown. *Graph* indicates the caspase-3 activity ratio for each condition (mean + SEM; n = 3; Mann–Whitney test; *P* < .05). (*J*) PP2A activity is increased by netrin-1 depletion and reversed by reduced expression of PR65β. *Graph* indicates PP2A activity ratio for each condition (mean + SEM; n = 3; Mann–Whitney test; *P* < .05). *,**, or *** refer to statistical analyses.

Netrin-1 is the ligand of the UNC5 DRs.^17^ To identify the receptor(s) responsible for caspase-3 activation in the absence of netrin-1, we first verified the expression of each netrin-1–receptor mRNA in HepaRG cells. As shown in Figure 7*A*, only *UNC5A* and *UNC5C* are expressed to detectable levels. Consequently, HepaRG cells were first transfected with each receptor’s siRNAs and then treated with DTT. Importantly, transfection of the individual siRNAs had no effect on XBP1 mRNA splicing and thus induction of the UPR (Figure 7*B*). Knockdown of netrin-1 protein was validated by immunoblotting (Figure 7*C*). Because of the lack of validated commercialized or in-house–generated antibodies, *UNC5A* and *UNC5C* silencing was verified by qRT-PCR. Their mRNA levels were decreased by at least 2-fold. No cross-reactivity between UNC5 siRNAs could be observed (Figure 7*D* and *E*). In line with our previous results, caspase-3 activity increased up to 25-fold in cells transfected with netrin-1 siRNA. Interestingly, double knockdown of netrin-1 and UNC5A or UNC5C rescued netrin-1 depletion-induced caspase-3 activation. Although UNC5A depletion led to a reduction of 60%, UNC5C completely reverted caspase-3 activation (Figure 7*F*). Altogether these results indicate that caspase-3 activation triggered by the UPR is mediated by UNC5A/C pro-apoptotic pathways in the absence of netrin-1.

DAPK1 recently was identified as a mediator of apoptosis in response to ER stress.^14^ The UNC5B pro-apoptotic pathway also activates DAPK1,^16^ suggesting a potential link between DAPK1 and UNC5A and UNC5C-conveyed signals during UPR. As before, HepaRG cells were transfected with control siRNA alone or with a combination of 2 siRNAs directed against netrin-1, DAPK1, or PR65β. UPR was verified by *XBP1* mRNA splicing (Figure 7*G*). Knockdown efficacies were assessed by immunoblotting for netrin-1, DAPK1, and PR65β (Figure 7*H*). As expected, netrin-1 depletion increased caspase-3 activity. Interestingly, this was partially and fully rescued by PR65β and DAPK1 knockdown, respectively (Figure 7*I*). To further corroborate the involvement of the DAPK1 pathway, we decided to also quantify the activity of PP2A, a DAPK1 phosphatase involved in the induction of apoptosis.^16^ The PP2A activity ratio was increased by 2-fold in cells transfected with netrin-1 siRNA. This activity returned to baseline upon depletion of PR65β, a PP2A regulatory subunit (Figure 7*J*). In summary, our results suggest that in hepatocytes, UPR-induced apoptosis is mediated by UNC5A and UNC5C receptors and involves the DAPK1/PP2A complex.^16^

### Netrin-1 Translation Is Resistant to UPR-Related Translational Shutdown in Mice Livers

**Figure 8:**
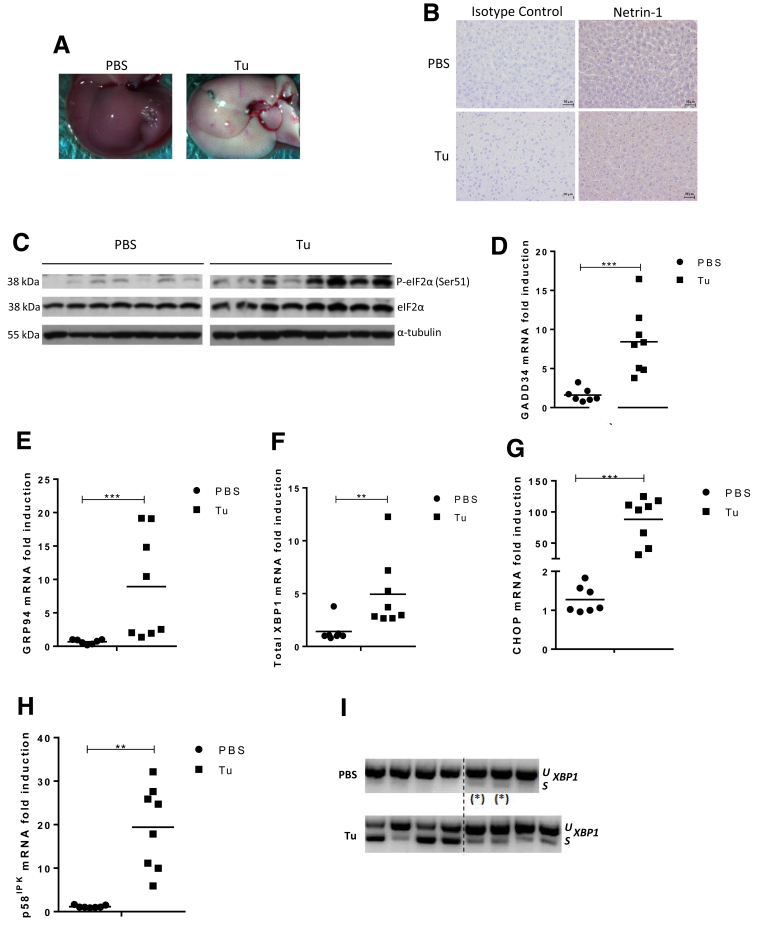
**Tunicamycin activates UPR in mice.** C57BL6 mice were treated with PBS or 1 mg/kg Tu and killed 24 hours after treatment. (*A*) Pictures from livers harvested 24 hours after treatment. Representative result, n = 7 (PBS), n = 8 (Tu). (*B*) Evaluation of netrin-1 protein overexpression by immunohistochemistry. Background level was assessed using an isotype control antibody and pictures were taken at a magnification of ×20. Representative result, n = 7 (PBS), n = 8 (Tu). (*C*) Evaluation of eIF2α phosphorylation level at Ser-51 (P-eIF2α [Ser-51]) by immunoblotting (n = 7 [PBS], n = 8 [Tu]). (*D–H*). Total RNA was extracted from mouse livers and *GADD34*, *GRP94*, total *XBP1*, *CHOP*, and *p58*^*IPK*^ mRNA levels were quantified by qRT-PCR (mean + SEM; n = 7 [PBS], n = 8 [Tu]; Mann–Whitney test; *P* < .05). (*I*) Assessment of XBP1 mRNA splicing by RT-PCR for each individual mouse. ^(^*^)^PBS-treated mice showing weak, intrinsic, XBP1 mRNA splicing. *,**, or *** refer to statistical analyses.

To test if our in vitro results could be verified in vivo, we treated C57BL6 wild-type mice with PBS or Tu for 24 hours using a published protocol.^56^ As previously shown, the livers of Tu-treated mice turned pale, indicating UPR-induced inhibition of liver function (Figure 8*A*).^56^ Immunohistochemistry showed that netrin-1 expression was insensitive to Tu (Figure 8*B*). Shutdown of protein translation was verified by considering the phosphorylation level of eIF2α at Ser-51 in Tu-treated mice (Figure 8*C*). *GADD34*, *GRP94*, total *XBP1*, *CHOP*, and *p58*^*IPK*^ mRNA levels were induced by 10-, 5-, 10-, 100-, and 20-fold, respectively, in treated vs control mice (Figure 8*D–H*). Induction of spliced *XBP1* mRNA in Tu-treated mice also was observed (Figure 8*I*). Taken together, these data suggest that the UPR is appropriately activated in these mice.

**Supplementary Figure 5:**
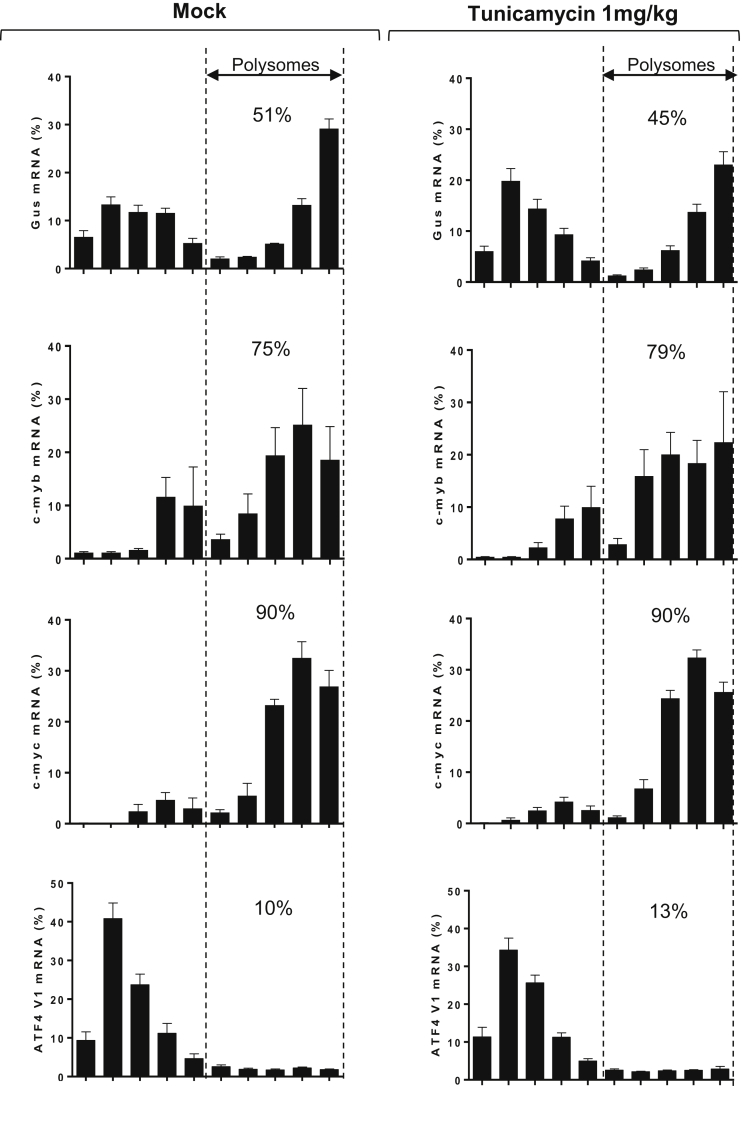
**Differential association of Netrin-1 related transcript with translational units during UPR in mice.** C57BL6 mice were treated with PBS or 1mg/kg Tunicamycin (Tu) and sacrificed 24 hours post-treatment. Livers were snap-frozen, disrupted in dry ice and then resuspended in polysome lysis buffer. Lysates were subjected to sucrose gradient fractionation followed by RT-qPCR to quantify gus, c-myb c-myc and ATF4 V1 mRNAs. Figure shows distribution of mRNAs across sucrose gradients. Bar graph represents RNA fractions processed for β-actin and netrin-1 mRNA quantification ((Mean + sem, n = 5 (PBS), n = 8 (Tu)). Agarose gel electrophoreses reflects ribosomal RNA distribution in the sucrose gradient. (Representative result, n + 5 (PBS), n + 8 (Tu)).

**Figure 9:**
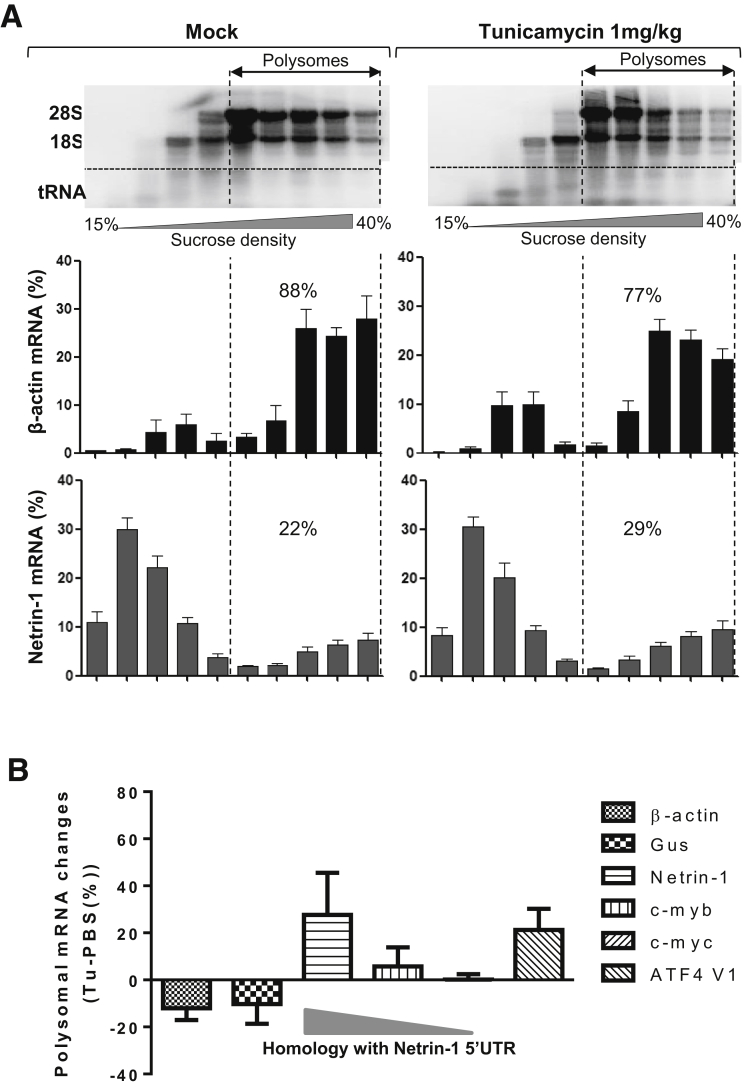
**Netrin-1 is positively selected for translation during UPR in mice.** C57BL6 mice were treated with PBS or 1 mg/kg Tu and killed 24 hours after treatment. Livers were snap-frozen, disrupted in dry ice, and then resuspended in polysome lysis buffer. Lysates were subjected to sucrose gradient fractionation followed by qRT-PCR to quantify *gus*, *β-actin*, *netrin-1*, *c-myb*, *c-myc*, and *ATF4* V1 mRNAs. (*A*) Distribution of mRNAs across sucrose gradients. *Bar graph* represents RNA fractions processed for *β-actin* and *netrin-1* mRNA quantification (mean + sem, n = 5 [PBS], n = 8 [Tu] for the entire figure). Agarose gel electrophoreses reflects ribosomal RNA distribution in the gradient. (*B*) The evolution of the association of indicated mRNAs with polysomes was determined as the percentage difference of polysome-associated mRNA signals in Tu samples vs mock samples in each polysome profile. See also Supplementary Figure 5.

Next, we performed polysome profiling of livers from treated or control mice to monitor netrin-1 translation in context of the UPR. *Netrin-1* profiles were compared with the same controls previously used in human cells, the only exception being *l-myc* mRNA, which was not detectable in mouse livers. Distribution of ribosomal RNAs after sucrose gradient fractionation indicated destabilization of polysomes by Tu as shown by agarose gel electrophoresis (Figure 9*A*, upper panel). Accordingly, *β-actin* mRNA association with polysomes was decreased from 88% to 77% after treatment, whereas *netrin-1* mRNA association with polysomes increased from 22% to 29% (Figure 9*A*, lower panels). This corresponded to a decrease in polysome association of 12% for *β-actin* mRNA, 10% for *GUS* mRNA, and to an increase of 27% for *netrin-1* and 21% for *ATF4* V1 mRNA (Figure 9*B*). As shown in vitro, polysome association of the control proto-oncogenes *c-myb* or *c-myc* transcripts decreased with their decreasing similarity with the *netrin-1* 5’UTR (Figure 9*B* and Supplementary Figure 5). In accordance with our in vitro results, netrin-1 translation even was affected positively by UPR-related translational shutdown in mice.

### Netrin-1 Inhibits UPR-Related Caspase-3 Activation in Mice Liver

To conclude this study, we wanted to determine whether netrin-1–overexpressing transgenic mice could provide further evidence for the protective role of netrin-1 during the UPR.

**Supplementary Figure 6:**
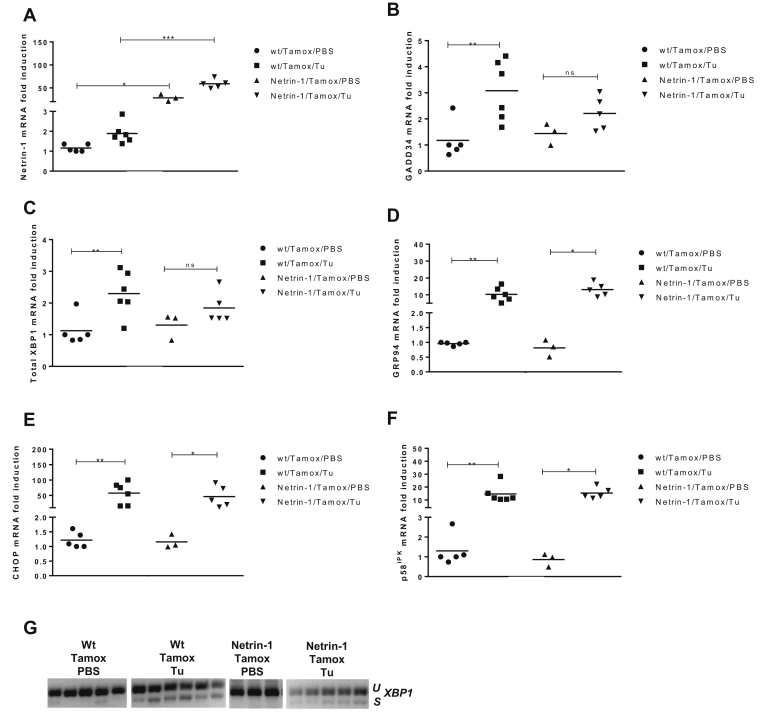
**Monitoring of UPR activation in Netrin-1 transgenic mice.** Netrin-1 transgenic mice or control littermates treated with Tamoxifen (Tamox) were injected with PBS or 1mg/kg Tu and sacrificed 24 hours post-treatment. Total RNA was extracted from mouse livers and subjected to RT-qPCR for quantification of Netrin-1 overexpression (*A*), GADD34 (*B*), total XBP1 (*C*), GRP94 (*D*), CHOP (*E*) and p58IPK (*F*) mRNAs. (Mean + sem, n = 5 (wt/tamox/PBS), n = 6 (wt/tamox/Tu), n = 3 (netrin-1/tamox/PBS), n = 5 (netrin-1/tamox/Tu), Mann-Whitney test, P<.05). (*G*) Assessment of XBP1 mRNA splicing by RT-PCR in each individual.

**Figure 10:**
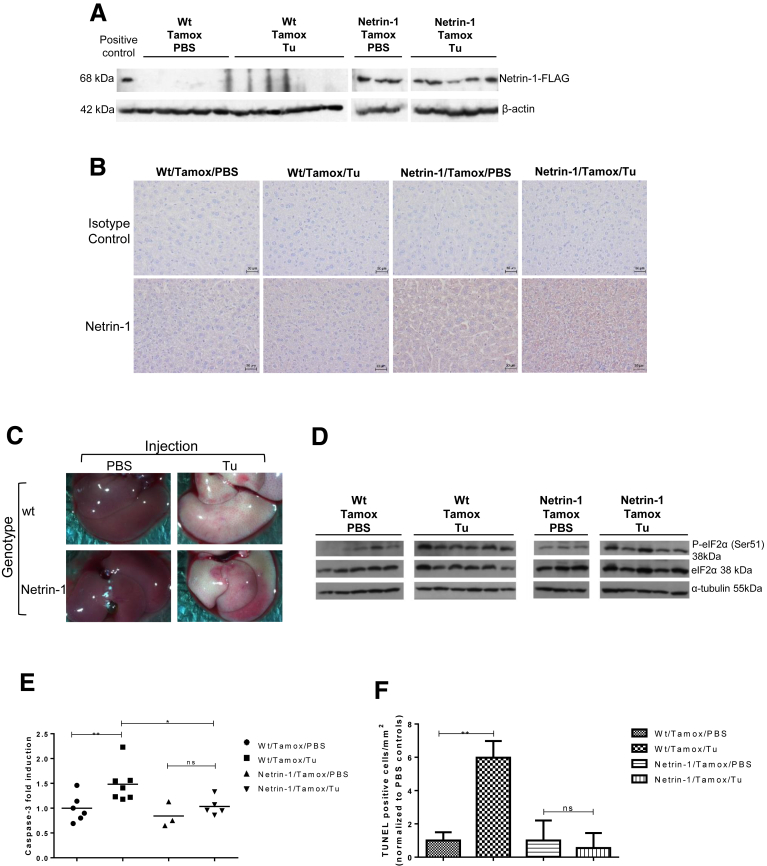
**Netrin-1 reverts UPR-induced caspase-3 activation in netrin-1 transgenic mice.** Netrin-1 (FLAG-tagged) transgenic mice or control littermates were treated with Tamoxifen (Tamox), injected with PBS, or 1 mg/kg Tu and killed 24 hours after treatment. (*A*) Evaluation of netrin-1 protein overexpression by anti-FLAG immunoblotting. (*B*) Evaluation of netrin-1 protein overexpression by immunohistochemistry. Representative result, n = 5 (wt/tamox/PBS), n = 6 (wt/tamox/Tu), n = 3 (netrin-1/tamox/PBS), n = 5 (netrin-1/tamox/Tu for the whole figure). Background level was assessed using an isotype control antibody. Magnification: ×20. (*C*) Liver pictures 24 hours after treatment. (*D*) Evaluation of eIF2α phosphorylation by immunoblotting. (*E*) Caspase-3 activation after Tu treatment is reversed in netrin-1–expressing transgenic mice. *Graph* indicates fold changes in caspase-3 activity compared with control mice (mean + SEM; Mann–Whitney test; *P* < .05). (*F*) The number of apoptotic cells is decreased in netrin-1 transgenic mice after Tu treatment. *Graph* indicates TUNEL positive cells/mm^2^ for each group (mean + SEM; Mann–Whitney test; *P* < .05). See also Supplementary Figure 6. *,**, or *** refer to statistical analyses.

Rosa-LSL-netrin-1 transgenic mice were crossed with Rosa-CreERT2+/+ mice to generate breeder pairs of control and conditional overexpressers of FLAG-tagged netrin-1. Animals were treated with tamoxifen to induce netrin-1 overexpression and then genotyped by RT-qPCR. *Netrin-1* mRNA was increased by 40- and 60-fold in transgenic mice compared with their wild-type counterparts when injected with PBS and Tu, respectively (Supplementary Figure 6*A*). Furthermore, anti-FLAG immunoblotting and immunohistochemistry confirmed netrin-1 overexpression (Figure 10*A* and *B*). As was the case in wild-type mice, liver of Tu-treated mice turned pale (Figure 10*C*). Again, Tu-treated mice showed induction of eIF2α phosphorylation at Ser-51 compared with their PBS-treated controls, indicating an attenuation of protein translation (Figure 10*D*). Activation of the PERK, ATF6, and IRE1α pathways by Tu were confirmed by induction of *GADD34* (3-fold), total *XBP1* (2-fold), *GRP94* (10-fold), *CHOP* (50-fold), and *p58*^*IPK*^ (15-fold) mRNA levels and *XBP1* mRNA splicing (Supplementary Figure 6*B*–*G*). We then analyzed the impact of netrin-1 on caspase-3 activity upon Tu treatment. As shown in Figure 10*E*, caspase-3 activity increased by 1.5-fold in control mice, but returned to baseline in netrin-1–overexpressing mice. In addition, TUNEL staining was performed by immunohistochemistry to quantify the increase of apoptotic cells. Although the number of TUNEL-positive cells was low in accordance with its end-stage apoptosis marker status, it was increased by 6-fold in control mice (Figure 10*F*). In contrast, TUNEL-positive cell numbers remained insensitive to Tu treatment in netrin-1 transgenic mice.

Altogether, these results suggest that netrin-1 protects against UPR-induced liver cell death in vivo.

## Discussion

After ER stress, cells activate a UPR to restore ER homeostasis, culminating in the PERK-mediated attenuation of protein synthesis. In this context, translation can occur through 2 distinct mechanisms. For example, the 5’UTR organization of the PERK-induced *ATF4* variant 2 mRNA allows its translation in a Cap-dependent manner. This is owing to a decreased translation of its 2 upstream Open Reading Frames, which are negative translation regulator elements.^57^ Intron retention in *ATF4* 5’UTR allows *ATF4* V1 mRNA transcription and translation in an IRES-dependent manner.^54^ Bioinformatic analysis showed a striking similarity between nucleotides 5 and 63 of *netrin-1* 5’UTR and the 5’UTR of *l-myc*, and between nucleotides 75 and 106 with the 5’UTR of *c-myb* mRNAs, both of which carry an IRES.^50^, ^51^ Polysome association of these transcripts increased along with their increasing similarity with the netrin-1 5’UTR. Moreover, *ATF4* V1 mRNA is associated strongly with polysomes during the UPR.^54^ These observations lead us to hypothesize that during UPR: (1) carrying an IRES is not enough for an mRNA to be translated efficiently, (2) a particular IRES folding seems to be required, and (3) *netrin-1* mRNA is translated in a cap-independent manner (netrin-1 5’UTR contains no uORF). This last point is supported further by the high guanine-cytosine content in the 5’UTR (>80%) of the transcript. In addition, the netrin-1 5’UTR was able to drive translation of an internal reporter cistron in a UPR intensity-dependent manner and efficiently recruited the 40S ribosomal subunit. Here, we show that in addition to the increased translation of several UPR-related targets such as *ATF4* V1^54^ and *CAT1* mRNA,^58^ netrin-1 translation is conserved or even stimulated in an IRES-dependent manner upon UPR-related translational shutdown.

RNA function is dependent on its architecture. Netrin-1 5’UTR structural analyses predicted a double pseudoknot formed by long-range interactions of the very 5’-end of the 5’UTR and the first nucleotides of the coding sequence. This element is a rare structural RNA motif previously described in viral and some cellular IRESs.^59^ In fact, it acts as the core nucleation center in the HCV IRES, mediating proper folding of each domain and the successful fitting of the resulting structure into the 40S ribosomal subunit.^60^ In addition, several so-called E-loops present throughout the entire *netrin-1* mRNA 5’-end were predicted. These are functional elements that promote the formation of accurately shaped scaffolds mediating protein recruitment and RNA–RNA interactions. Taken together, our functional and structural data provide strong support for the existence of an IRES element in the *netrin-1* 5’UTR. The translational induction of netrin-1 during hypoxia, during which cap-dependent translation is attenuated, further corroborates our results.^61^

In this study, we also show that netrin-1 plays a protective role in experimentally induced UPR, both in vitro and in vivo. UNC5A or UNC5C knockdown reduces caspase-3, suggesting that these dependence receptors mediate apoptosis during the UPR. There are no existing data on UNC5C pro-apoptotic pathway whereas UNC5A is known to recruit NRAGE for apoptosis induction.^62^ However, UNC5B is known to trigger apoptosis via PP2A-mediated dephosphorylation of DAPK1.^16^ In this study, silencing of PR65β, the regulatory subunit of PP2A, was sufficient to inhibit UNC5A and UNC5C-mediated apoptosis, suggesting the involvement of DAPK1. Because PP2A and DAPK1 have been shown previously to be involved in UPR-induced apoptosis,^14^ we investigated a possible link between DAPK1 and the downstream signaling of UNC5A and UNC5C during UPR-induced apoptosis. Our data suggest the following: (1) netrin-1 could protect against UPR-induced cell death by inhibiting pro-apoptotic pathways induced by free UNC5A and UNC5C receptors, and (2) UNC5A and UNC5C trigger apoptosis after recruitment of PP2A and DAPK1 activation.

On a broader pathophysiological note, hepatocellular carcinoma (HCC) constitutes a serious global medical challenge. Several chronic liver conditions leading to fibrosis and cirrhosis, which can in turn foster HCC onset, are associated with ER stress.^5^, ^12^ For instance, α1-antitrypsin deficiency is sensitized to ER stress because of the misfolding of this protein.^63^ Likewise, alcoholic liver disease and infection with HBV or HCV are strong inducers of UPRs.^7^, ^8^, ^9^, ^30^, ^31^, ^32^, ^64^, ^65^ Even successfully treated HBV patients still experience a chronic UPR because of the unchanged amount of secreted defective subviral material that burdens the secretory pathway of the affected hepatocytes.^66^ Activation of ER stress-induced signaling is in turn instrumental for the development of steatohepatitis and synergizes with proinflammatory pathways to promote hepatocarcinogenesis.^67^ It is believed that cirrhosis imposes a high functional burden to the ER of the remaining liver cells, in which increased production of plasma proteins takes place to compensate for the loss of hepatocytes as a result of liver damage. Hence, the UPR also may promote cell survival during cirrhosis.^7^ The UPR is a typical prerequisite for cancer development, namely in highly secretory cell types. HCC is a strongly secretory tumor type, which may in turn rely on this secretory addiction to sustain transformation through the induction of the UPR.^68^ In this work, we have shown that the UPR makes cells more dependent toward netrin-1 for survival, and netrin-1 may be angiogenic.^69^ The UPR contributes to tumor growth and angiogenesis, enhancing the survival of cancer cells to the hypoxic and low-nutrient conditions observed in solid tumors such as HCC. Sorafenib, the approved targeted therapy against HCC,^70^ fosters HCC cell death through antiangiogenic activity^71^ and inhibition of the UPR.^72^, ^73^, ^74^ It is possible that, as is the case for any dynamic process in biology, dual targeting of the UPR using sorafenib and netrin-1 in HCC may decrease the dependency of HCC cells toward netrin-1 for cell survival. Nevertheless, one can in contrast hypothesize that potentially lower rates of secreted netrin-1 as a result of sorafenib treatment may improve netrin’s neutralization rates and therefore further affect global survival of the tumor.

In summary, several chronic liver conditions, regardless of being genetic, viral, or toxicologic in origin, feature enhanced UPRs. Netrin-1 currently is targeted in phase 1 trials in nonhepatic oncology. Although dual UPR/netrin-1–targeting approaches deserve preclinical investigations, they may represent a hitherto unknown and innovative option for addressing HCC onset and growth at the cirrhotic level.

## Figures and Tables

**Supplementary Table 1:** Human Primers

Gene name	Genbank accession number	Primer sequences	PCR conditions	Amplicon length, *bp*
Netrin-1	NM_004822	Forward: CTTCTGCGGCAGGCGGACAGAT; reverse: ACGCGTTGCAGAGGTGGCACGA	Denaturation, 95°C; annealing, 65°C (qPCR with 10% DMSO)	385
Gus	NM_001293105	Forward: CGTGGTTGGAGAGCTCATTTGGAA; reverse: TTCCCCAGCACTCTCGTCGGT	Denaturation, 95°C; annealing, 60°C	72
β-actin	XM_006715764.1	Forward: CACCATTGGCAATGAGCGGTTC; reverse: AGGTCTTTGCGGATGTCCACGT	Denaturation, 95°C; annealing, 60°C	135
p58^IPK^	NM_006260.4	Forward: CGTTCACAAGCACTTAACGCT; reverse: GTTCTGCATCCCAAACACAAAC	Denaturation, 95°C; annealing, 55°C	97
GADD34	NM_014330.3	Forward: ATGATGGCATGTATGGTGAGC; reverse: AACCTTGCAGTGTCCTTATCAG	Denaturation, 95°C; annealing, 55°C	120
GRP94	NM_003299.2	Forward: CCCCATGGCTTATATTCACTTTAC; reverse: TCTTGTTTTTCCTTCATTCTTTCC	Denaturation, 95°C; annealing, 60°C	504
CHOP	NM_004083.5	Forward: AAGGCACTGAGCGTATCATGT; reverse: TGAAGATACACTTCCTTCTTGAAC	Denaturation, 95°C; annealing, 60°C	105
Total Xbp1	NM_005080.3	Forward: CCCTCCAGAACATCTCCCCAT; reverse: ACATGACTGGGTCCAAGTTGT	Denaturation, 95°C; annealing, 55°C	101
Xbp1	NM_005080.3 NM_001079539.1	Forward: GGAACAGCAAGTGGTAGA; reverse: CTGGAGGGGTGACAAC	Denaturation, 95°C; annealing, 60°C	259/233
l-myc	NM_001033081.2	Forward: CCATCAGCAACAGCACAACTA; reverse CACTTTCTACAGGTGGGGGA	Denaturation, 95°C; annealing, 55°C	167
c-myb	XM_006715495.1	Forward: GAAAGCGTCACTTGGGGAAAA; reverse: TGTTCGATTCGGGAGATAATTGG	Denaturation, 95°C; annealing, 55°C	122
c-myc	NM_002467.4	Forward: ACCACCAGCAGCGACTCTGA; reverse: TGCCAGGAGCCTGCCTCTTT	Denaturation, 95°C; annealing, 55°C	85
ATF4 V1	NM_001675.4	Forward: CCACCATGGCGTATTAGGGG; reverse: GCACCCCACCAATCCAAAAC	Denaturation, 95°C; annealing, 55°C	177
DAPK1	NM_001288731.1	Forward: ACGTGGATGATTACTACGACACC; reverse: TGCTTTTCTCACGGCATTTCT	Denaturation, 95°C; annealing, 57°C	81
PR65β	NM_001161660.1	Forward: CTTGGCGCGTTCGCTATATG; reverse: GGCGGGGATGAGGTCATTTAG	Denaturation, 95°C; annealing, 57°C	92
UNC5A	XM_006714928.1	Forward: CATCACCAAGGACACAAGGTTTGC; reverse: GGCTGGAAATTATCTTCTGCCGAA	Denaturation, 95°C; annealing, 57°C	125
UNC5B	NM_170744.4 NM_001244889.1	Forward: GGGCTGGAGGATTACTGG; reverse: TGCAGGAGAACCTCATGGTC	Denaturation, 95°C; annealing, 60°C	155
UNC5C	NM_003728.3	Forward: GCAAATTGCTGGCTAAATATCAGGAA; reverse: GCTCCACTGTGTTCAGGCTAAATCTT	Denaturation, 95°C; annealing, 60°C	114
UNC5D	NM_080872.2	Forward: GGTGAACCCAGCCTCCAGTCAG; Reverse: CTTCCACTGACATCACTTCCTCCC	Denaturation, 95°C; annealing, 60°C	199
DCC	XM_005258204.1	Forward: AGCCAATGGGAAAATTACTGCTTAC; reverse: AGGTTGAGATCCATGATTTGATGAG	Denaturation, 95°C; annealing, 60°C	126

DMSO, dimethyl sulfoxide.

**Supplementary Table 2:** Mouse Primers

Gene name	Genbank accession number	Primer sequences	PCR conditions	Amplicon length, *bp*
Mouse β-actin	NM_007393.3	Forward: GCAGGAGTACGATGAGTCCG; reverse: ACGCAGCTCAGTAACAGTCC	Denaturation, 95°C; annealing, 55°C	74
Mouse Gus	NM_010368.1	Forward: GTGGTATGAACGGGAAGCAAT; reverse: AACTGCATAATAATGGGCACTGT	Denaturation, 95°C; annealing, 55°C	97
Mouse netrin-1	NM_008744.2	Forward: CCTGTCACCTCTGCAACTCT; reverse: TGTGCGGGTTATTGAGGTCG	Denaturation, 95°C; annealing, 55°C	78
Mouse GADD34	NM_008654.2	Forward: CCCAGCGTTGTCTACCAGG; reverse: CGGGTAAATAGAAGGCCACCT	Denaturation, 95°C; annealing, 55°C	81
Mouse GRP94	NM_011631.1	Forward: GTTCGTCAGAGCTGATGATGAA; reverse: GCGTTTAACCCATCCAACTGAAT	Denaturation, 95°C; annealing, 55°C	138
Mouse CHOP	NM_007837.4	Forward: AAGCCTGGTATGAGGATCTGC; reverse: TTCCTGGGGATGAGATATAGGTG	Denaturation, 95°C; annealing, 55°C	75
Mouse P58^IPK^	NM_008929.3	Forward: CACCAAAGTGATTGCGCTGAA; reverse: ATCGTCTTCTGCTTCGTCAAG	Denaturation, 95°C; annealing, 55°C	100
Mouse total Xbp1	NM_001271730.1	Forward: AGCTTTTACGGGAGAAAACTCAC; reverse: CCTCTGGAACCTCGTCAGGA	Denaturation, 95°C; annealing, 55°C	99
Mouse Xbp1	NM_001271730.1 NM_013842.3	Forward: AGCAGCAAGTGGTGGA; reverse: CTGGGGAGGTGACAAC	Denaturation, 95°C; annealing, 60°C	263/237
Mouse c-myb	NM_001198914.1	Forward: GAAAGCGTCACTTGGGGAAAA; reverse: TGTCCGGTTGGGCAGATAATTGG	Denaturation, 95°C; annealing, 55°C	122
Mouse c-myc	NM_001177353.1	Forward: ACCACCAGCAGCGACTCTGA; reverse: TGGCAGGGGTTTGCCTCTTC	Denaturation, 95°C; annealing, 55°C	85
Mouse ATF4 V1	NM_009716.3	Forward: CCGGAAATTCGTCAACGAGC; reverse: AGATCGTCCTAAAGGCCCCA	Denaturation, 95°C; annealing, 54°C	109

**Supplementary Table 3:** Other Primers

Gene name	Genbank accession number	Primer sequences	PCR conditions	Amplicon length, *bp*
Netrin-1 (5’UTR 1-294)	NM_004822	Forward: (T7p5’UTR_*Netrin-1)* TAATACGACTCACTATAGCTTCGGGGGCGAGC; reverse: (cas-as5’UTR_Netrin-1) GAACCGGACCGAAGCCCGATTTGGATCCGGCG AACCGGATCGACCTTGCCGAAGGCCGCAT	Denaturation, 95°C; annealing, 65°C (qPCR with 10% DMSO)	294
/	/	Forward: (primer Std) GAACCGGACCGAAGCCCG	/	/
Fluc	/	Forward: TGTCCACCTCGATATGTGCATC	/	/

/ signify that there is no gene name, PCR conditions and amplicon length for these primers. They were used as labeled probes. DMSO, dimethyl sulfoxide.

**Supplementary Table 4:** siRNA Sequences

Target gene	siRNA sequence 5’-3’	Source
Control	Commercial	SIC001 (Sigma)
Netrin-1	AAGCUGGACGCAGCAUGAUGC	Sigma
UNC5A	CUGGACACCCGCAACUGUA	Sigma
UNC5C	CUCUAUGUUGGGAUUGUGA	Sigma
DAPK1	Commercial	EHU015411 (Sigma)
PR65β	Commercial	EHU059311 (Sigma)

**Supplementary Table 5:** Antibody Information

Target protein	Name	References
HA-Tag	Monoclonal anti-HA antibody	H3663 (Sigma)
FLAG-M2	Monoclonal anti-FLAG M2 antibody	F3165 (Sigma)
Netrin-1	Polyclonal anti–netrin-1 antibody	AF1109 (R&D Systems)
Netrin-1	Polyclonal anti–netrin-1 antibody	MAB1109 (R&D Systems)
β-actin	Polyclonal anti–β-actin antibody	A1978 (Sigma)
DAPK1	Monoclonal anti-DAPK1 antibody	D2178 (Sigma)
PR65β	Monoclonal anti-PP2R1B antibody	Ab154815 (Abcam)
eIF2α	Polyclonal anti-eIF2α antibody	9722 (Cell Signaling)
Phospho-eIF2α (Ser-51)	Polyclonal anti–phospho-eIF2α (Ser51) antibody	9721 (Cell Signaling)
α-tubulin	Polyclonal anti–α-tubulin antibody	PA1-20988 (Thermo Scientific)
